# Implementability of healthcare interventions: an overview of reviews and development of a conceptual framework

**DOI:** 10.1186/s13012-021-01171-7

**Published:** 2022-01-27

**Authors:** Marlena Klaic, Suzanne Kapp, Peter Hudson, Wendy Chapman, Linda Denehy, David Story, Jill J. Francis

**Affiliations:** 1 The University of Melbourne, School of Health Sciences, Melbourne, Australia; 2 The Royal Melbourne Hospital, Allied Health Department, Melbourne, Australia; 3 The University of Melbourne, School of Health Sciences, Faculty of Medicine, Dentistry and Health Sciences, Department of Nursing, Melbourne, Australia; 4 Centre for Palliative Care, St Vincent’s Hospital, Melbourne, Australia; 5 End-of-life Care Research Department, Vrije Universiteit Brussel (VUB), Brussels, Belgium; 6 Centre for Digital Transformation of Health, The University of Melbourne, Melbourne, Australia; 7 Department of Allied Health, Peter McCallum Cancer Centre, Melbourne, Australia; 8 Department of Critical Care, The University of Melbourne, Melbourne, Australia; 9 Department of Anaesthesia, Austin Health, Melbourne, Australia; 10 Ottawa Hospital Research Institute, Clinical Epidemiology Program, Ottawa, Canada; 11 Department of Health Services Research, Peter McCallum Cancer Centre, Melbourne, Australia

**Keywords:** Implementation strategies, Framework, Scalability, Sustainability, Implementability, Implementation science, Implementation research, Healthcare interventions

## Abstract

**Background:**

Implementation research may play an important role in reducing research waste by identifying strategies that support translation of evidence into practice. Implementation of healthcare interventions is influenced by multiple factors including the organisational context, implementation strategies and features of the intervention as perceived by people delivering and receiving the intervention. Recently, concepts relating to perceived features of interventions have been gaining traction in published literature, namely, acceptability, fidelity, feasibility, scalability and sustainability. These concepts may influence uptake of healthcare interventions, yet there seems to be little consensus about their nature and impact. The aim of this paper is to develop a testable conceptual framework of implementability of healthcare interventions that includes these five concepts.

**Methods:**

A multifaceted approach was used to develop and refine a conceptual framework of implementability of healthcare interventions. An overview of reviews identified reviews published between January 2000 and March 2021 that focused on at least one of the five concepts in relation to a healthcare intervention. These findings informed the development of a preliminary framework of implementability of healthcare interventions which was presented to a panel of experts. A nominal group process was used to critique, refine and agree on a final framework.

**Results:**

A total of 252 publications were included in the overview of reviews. Of these, 32% were found to be feasible, 4% reported sustainable changes in practice and 9% were scaled up to other populations and/or settings. The expert panel proposed that scalability and sustainability of a healthcare intervention are dependent on its acceptability, fidelity and feasibility. Furthermore, acceptability, fidelity and feasibility require re-evaluation over time and as the intervention is developed and then implemented in different settings or with different populations. The final agreed framework of implementability provides the basis for a chronological, iterative approach to planning for wide-scale, long-term implementation of healthcare interventions.

**Conclusions:**

We recommend that researchers consider the factors acceptability, fidelity and feasibility (proposed to influence sustainability and scalability) during the preliminary phases of intervention development, evaluation and implementation, and iteratively check these factors in different settings and over time.

**Supplementary Information:**

The online version contains supplementary material available at 10.1186/s13012-021-01171-7.

Contributions to the literature

- Reviews report relatively few healthcare interventions that are sustained beyond the initial implementation phase or scaled to different populations or settings.
- Acceptability, fidelity and feasibility may influence scalability and sustainability of a healthcare intervention.
- We have developed a testable conceptual framework that can be used to prospectively and iteratively guide the implementability of healthcare interventions.
- Prospective identification of factors that influence scalability and sustainability of a healthcare intervention is critical to avoid or reduce research waste.

## Background

Implementation science aims to identify and address care gaps, support practice change and enhance quality and equity of health care. Building a robust and generalizable evidence base to inform implementation practice is the objective of implementation research. Implementation research can also play a critical role in efforts to reduce research waste, in that it can provide evidence about the strategies that are effective for translating the findings of clinical research into enhanced healthcare practice and thus improved health outcomes [1–3]. Identifying the factors important for translation of an effective intervention or innovation from the research setting to routine clinical practice can arguably contribute to reducing the estimated annual US$85 billion, globally, wasted in health research [1, 2, 4].

Most implementation investigations focus on one of two approaches to achieve change. First, implementation activities consist of either “top down” processes (e.g. governance arrangements, national policies and guidelines, continuing medical education, incentivisation systems) [5–7], or more granular “bottom-up” processes that consider views of healthcare workers: their perceived barriers and enablers to specific elements of practice change at the level of healthcare teams and individual clinicians [8–11]. The second approach considers features of healthcare contexts (including organisational factors and the wider health system context) that might interact with the implementation activities to enable or impede practice change [12–16].

The current paper considers a third lever for achieving implementation: the perceived features of healthcare interventions themselves (in addition to effectiveness). An early theory, Diffusion of Innovations [17], identified six features of innovations that make their adoption more or less likely, namely, relative advantage, compatibility with the existing system, complexity, trialability, potential for reinvention and observed effects, where trialability refers to being able to test the innovation or intervention on a small scale, such as a pilot study. The more recent Consolidated Framework of Implementation Research (CFIR) proposed seven attributes of interventions, namely, intervention source, evidence strength and quality, relative advantage, adaptability, trialability, complexity and design quality and packaging, which refers to the presentation of the intervention, such as how it is bundled and user accessibility [12]. A recently published review identified 28 implementation frameworks and models, including the CFIR, which were synthesised into a number of core phases and components [18]. The authors suggest there is a need for an overarching framework that can guide researchers from intervention development to sustainable practice change.

**Fig. 1:**
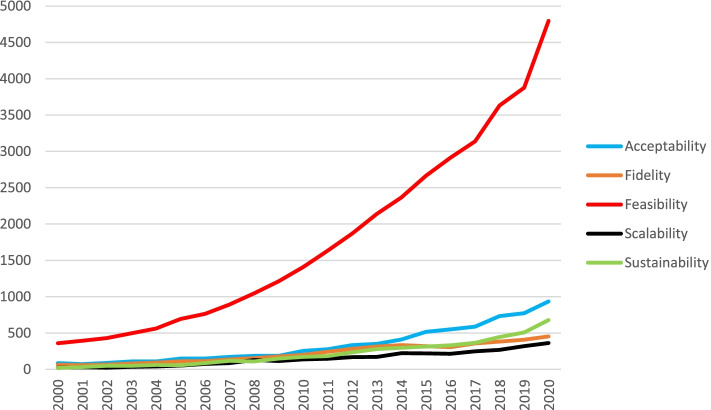
Annual frequency of the five key concepts in publications indexed to PubMed

Uptake of an intervention by both providers and recipients also depends crucially on their perceptions of the intervention. The COVID pandemic of 2020–2021 exemplifies this point. Even though approved vaccine interventions have substantial evidence of a positive benefit-to-risk ratio, the speed of uptake in many countries has been dependent on the perceptions of politicians, service providers and members of the public regarding the necessity, urgency and benefits of vaccine programs. It seems that, independent of the objective features of an intervention, stakeholder perceptions about the intervention will radically influence implementation and uptake at many levels. Furthermore, these perceptions may change over time and during roll-out of an intervention. We refer to these perceptions as views about the “implementability” of an intervention. We define “implementability” as the likelihood that an intervention will be adopted into routine practice and into health consumer behaviours across settings and over time. Several concepts related to implementability of healthcare interventions are gaining traction in the implementation science literature and appear to be primarily focused on the earlier stages of intervention development or latter stages of evaluation. These are acceptability, fidelity, feasibility, scalability and sustainability. A search of the health sciences literature (conducted 25th February 2021) for studies published in the last 20 years containing one or more of these five concepts in the title, revealed that the annual frequency of usage has steadily increased. Figure 1 shows that, from a relatively low baseline in the year 2000, these concepts appeared in titles in 2020, respectively, > 900, > 450, > 4500, > 350 and > 650 times.

Where there was an explicit rationale in the included studies, authors noted that the concept under investigation was likely to influence engagement, adoption and ongoing use. For example, a systematic review exploring videoconferencing in an orthopaedic setting [19] found that “acceptability of service users (both patients and clinicians) is a key factor for the uptake of telemedicine in clinical practice” (p.184). Hence, it is plausible that these concepts, individually and collectively, influence intervention implementability. To identify whether other implementation-related concepts were also appearing in the literature, we conducted a further illustrative search of literature published in the last 20 years (conducted on 29th August 2021) relating to specific interventions (using the phrase “of [intervention]”). We selected three healthcare interventions for which there were published reviews including one or more of the five concepts. The majority (> 90%) of reviews focused on clinical effectiveness or evaluation of outcomes. No additional concepts related to implementability, other than the five considered in our proposed framework, were evident.

There seems to be little consensus about the nature of these concepts, appropriate measurement strategies and how they might be related to one another. Without consistent definitions or reliable measurement approaches, it is not possible to test assumptions or predictions about whether these features indeed influence the implementability of healthcare interventions.

Implementability has been previously explored in the published literature, but this has focused on the implementability of clinical practice guidelines [20–23] or, more recently, the implementability of late-phase clinical trials [24]. The current paper focuses more broadly on prospective implementability of healthcare interventions, particularly at the early stages of development and evaluation, during scale-up, and over time.

The aim of this paper is to report the development of a testable conceptual framework of implementability that includes acceptability, fidelity, feasibility, scalability and sustainability.

## Methods

A multifaceted approach was used to develop and refine the framework of implementability of healthcare interventions. We use the World Health Organization definition of healthcare interventions as “an act performed for, with or on behalf of a person or population whose purpose is to assess, improve, maintain, promote or modify health, functioning or health conditions.” [25]

### Step 1: Overview of reviews

A preliminary exploratory search indicated a large volume of systematic reviews on the aforementioned five concepts within published literature on healthcare interventions. We therefore decided to conduct an overview of reviews [26], to answer the following questions:

Have the five concepts (acceptability, fidelity, feasibility, scalability and sustainability) been defined, operationalised and/or theorised in systematic reviews (SR) on healthcare interventions?

Have the five concepts been combined in any publications, frameworks or models used in published literature on healthcare interventions?

#### Search strategy

Systematic reviews published from January 2000 to March 2021 were identified and retrieved by one author (MK). Searches were structured by combining relevant review filters (Additional file 1: search strategy), with the appearance of the truncated term for each concept (“acceptab*”, “fidelity”, “feasib*”, “sustainab*” and “scalab*”) in article titles. Searching for these terms in article titles ensured that the review had a primary focus on the concept. The term feasibility was combined with “or process evaluat*” but papers were considered for inclusion only if the term feasibility was in the abstract. We did not include a synonym search as we were specifically interested in the use of the particular concept terms in the literature.

Multiple databases were searched using OVID (Medline and Embase) and EBSCO (PsycINFO) and restricted to publications in English.

#### Screening citations

Duplicates were removed using the deduplicate option in the OVID and EBSCO search engines and the remaining citations were imported into EndNote X9 [27] where further duplicates were manually identified and removed. Reviews were considered eligible for inclusion if they met the criteria detailed in Table 1. Screening was a two-step process, commencing with an initial review of the abstracts by one author (MK) to determine eligibility. If there was insufficient information in the abstract to make a decision regarding inclusion, the full paper was retrieved, and the methods section was screened. For example, drug-development reviews with acceptability or feasibility in the title but a focus on cost-effectiveness acceptability curves were excluded.

**Table 1:** Inclusion and exclusion criteria for the overview of reviews

Inclusion criteria	Exclusion criteria
Systematic review	Not available in English
Healthcare intervention	Focus on cost-effectiveness acceptability curves
Inclusion of acceptability, fidelity, feasibility, scalability and/or sustainability in the title	Reviews on drug development where the focus was only on safety
	Conference proceedings, commentaries, study protocols and editorials

#### Full-text review

The full articles for all citations that met the inclusion criteria were retrieved by one author (MK) with an additional author (SK) independently reviewing a random selection of 10% of the retrieved papers. Data were extracted using a form developed by the research group and included if and how the concept was defined, how it was operationalised or measured, use or development of a framework and overall outcome of the review, i.e. had the healthcare intervention achieved acceptability, fidelity, feasibility, scalability or sustainability? A review was considered to have used a framework if it was mentioned in the methods and data were synthesised using the components of the stated framework. Primary studies included in the reviews were not individually reviewed as this was considered to be outside of the scope of this overview. Data extracted from the reviews were summarised descriptively.

#### Assessment of quality

Assessment of quality was not conducted as the aim of the study was to explore how the implementability concepts were defined and conceptualised, rather than the quality of the information related to the health intervention being delivered.

### Step 2: Development of the preliminary framework

**Fig. 2:**
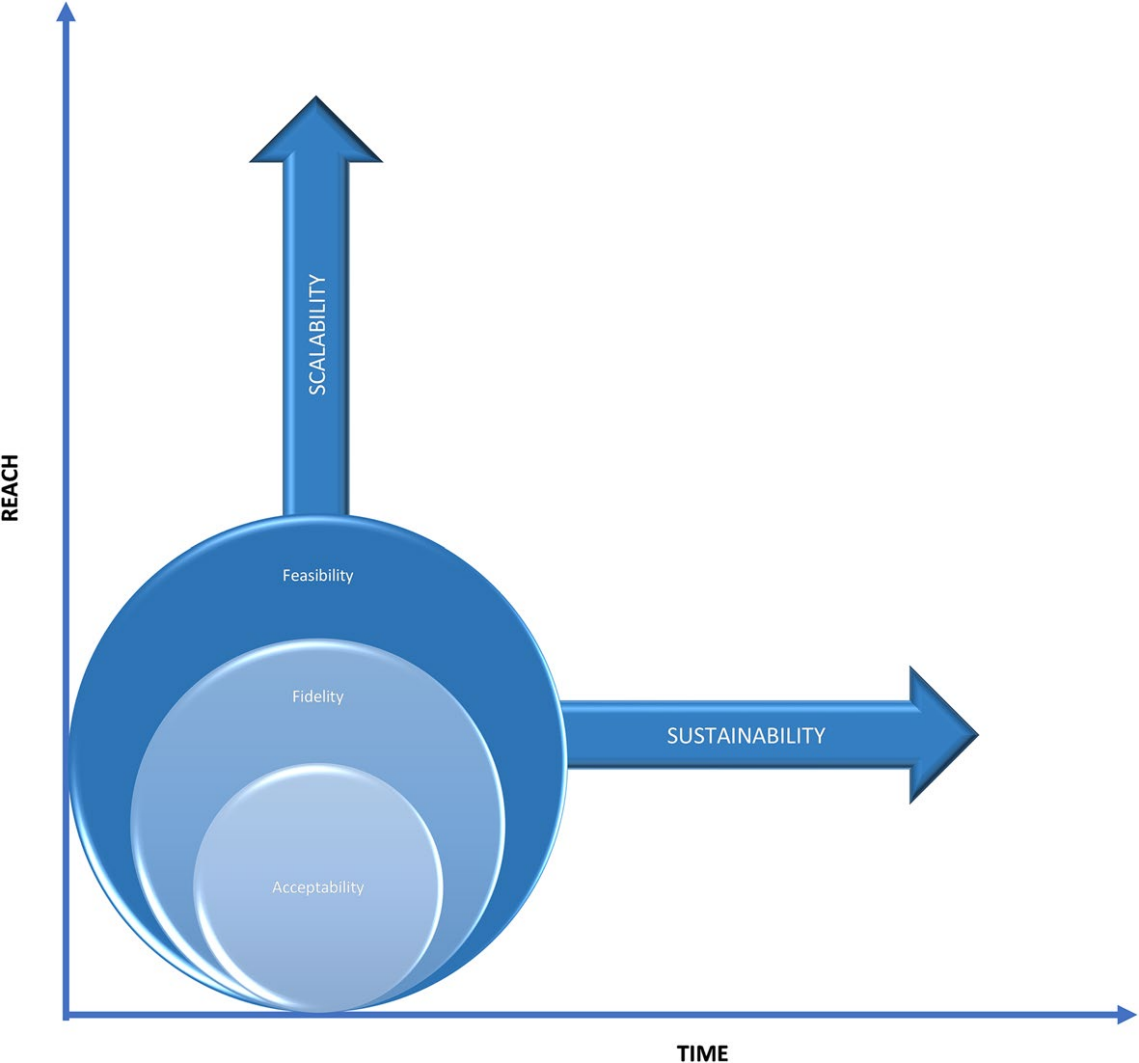
Initial framework of implementability of healthcare interventions

A preliminary framework (Fig. 2) was developed in parallel with the overview of reviews, integrating the five concepts that appeared to be mostly investigated in isolation in the published reviews on healthcare interventions, and based on the research experiences and theory-development expertise of two authors (JF and MK). This framework was then presented to the group of experts (co-authors), as described below.

### Step 3: Modified Nominal Group Technique

The Nominal Group Technique (NGT) is a facilitator-led, structured process for obtaining information and arriving at a decision with a target group who have some association or experience with the topic [28]. Various adaptations of the NGT have been used in conceptual studies that focus on framework development [29–33]. Recently, an additional pre-meeting, information-giving step has been suggested to enable more time for participants to consider their contribution to the topic [34, 35]. The adapted NGT process utilised in this study was as follows: (i) identification of group members, to include experts with depth and diversity of experience [36]. All authors on this paper were invited by e-mail to attend an online group meeting. They were purposively identified at the start of this study for their knowledge and expertise in the fields of implementation science, theory development, biomedical informatics and clinical research across a broad range of fields; (ii) provision of information prior to the meeting, including a PowerPoint presentation, findings of the overview of reviews and objectives of the meeting. Five authors with extensive clinical research backgrounds were asked to prepare a clinical scenario on one concept for sharing at the group meeting. The intention of this exercise was to discuss the fit between a real-world example of a study that explored one of the concepts and the proposed framework; (iii) meeting conducted online and facilitated by one author (JF) who has extensive experience in consensus panel processes. Following presentation of the meeting materials, including the preliminary framework, group members were instructed to silently consider the framework and generate ideas and critiques; iv) round-robin process with participants sharing their ideas and critiques; v) clarification process where participants shared their clinical scenario on a concept and discussed the fit with components of the initial framework, and vi) voting and/or agreement on the preliminary framework.

## Results

### Step 1: Overview of reviews

**Fig. 3:**
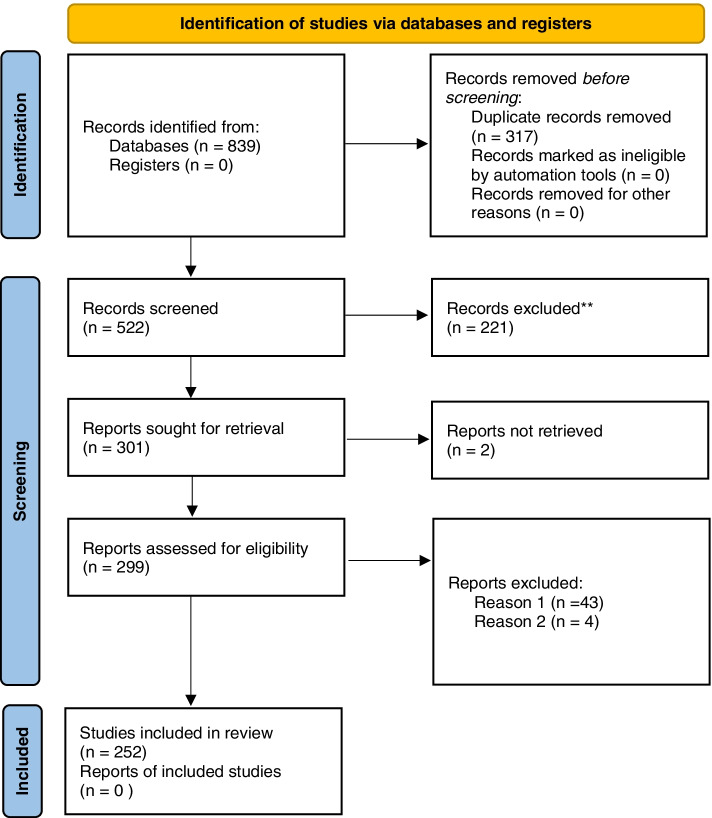
PRISMA [37] flow chart of included reviews for search completed in March 2021

The database searches initially identified a total of 839 references across all five concepts (acceptability = 224, fidelity = 281, feasibility = 253, scalability = 37 and sustainability = 44). Following removal of 317 duplicates and screening of titles and abstracts, 301 full texts were sought for retrieval. Two were not retrieved as they were not available in English. Of the remaining 299 reports assessed for eligibility, 43 were excluded due to being unrelated to the concept (e.g. fidelity of DNA) and four were excluded as they focused on psychometric testing of a measure rather than a health intervention. The final number of publications included in this review was 252, of which 22 papers discussed more than one concept. As we were considering the concepts separately, these 22 were treated as separate investigations, resulting in a total of 274 investigations (Additional file 2: reviews included in the overview, consisting of acceptability = 132, fidelity = 41, feasibility = 65, scalability = 11 and sustainability = 25), with the stages of the search process presented in Fig. 3.

#### Characteristics of the included studies

Of the 252 studies in the overview, 30% included a meta-analysis and 19% used a mixed-methods approach that incorporated both quantitative and qualitative data from empirical research. The healthcare interventions that were a focus of the review were broad ranging and included psychological/psychiatric/psychosocial interventions (20%), technology-based interventions (5%), physical activities (6%), pharmacological and alternative interventions. The number of studies included in reviews ranged from 4 to 296, with the majority of studies (63%) having no setting exclusions (or not reported). Acceptability and fidelity were assessed using highly variable measurement approaches, so it was not appropriate to summarise the findings across reviews. The intervention under investigation was reported to be feasible in 32 (49%) reviews, sustainable in 1 (4%) review and successfully scaled up in 1 (9%) review (Additional file 3).

#### Definition and measurement (question a) and frameworks (question b) for the five concepts

A total of 1096 items of information were extracted from the 274 investigations by the first reviewer (MK). The second reviewer (SK), double extracted information from 10% of the reviews (total 32 papers double reviewed), which were compared with the first reviewer to assess reliability.

Of these, 100% agreement was achieved for definition of the concept and outcome of the review (e.g. acceptability or fidelity or feasibility or scalability or sustainability was/was not achieved). There was 96% agreement on the use of a framework and 89% agreement on the constructs measured. One reviewer failed to identify a framework in one of the studies resulting in 96% (out of 32 studies double reviewed) agreement. For constructs measured, reviewers did not identify the same constructs for two papers, resulting in 89% agreement. The two reviewers discussed these differences and agreed on a decision.

Twenty-two publications included two concepts in their review, of which 20 considered acceptability and feasibility [38–57] in exploring implementation of a healthcare intervention, and two considered scalability and sustainability [58, 59].

#### Acceptability

Of the reviews exploring acceptability of a healthcare intervention, only 13 provided an *a priori* definition and they largely focused on whether those receiving a healthcare intervention found it to be “appropriate” and “fair” and “reasonable” [42, 48, 60–66]. Four reviews considered acceptability from the perspective of those delivering a healthcare intervention [61, 62, 65, 66].

The majority of reviews measured one variable in evaluating acceptability, and this was predominantly either dropout rates (33%) or user perceptions of the intervention (30%). Twenty-three reviews measured three or more component variables, most commonly a combination of participant dropouts, recruitment rates, perceptions of users such as satisfaction measures, adherence to the study protocol and adverse events (3%).

Six reviews used a framework to define and measure acceptability, as described in Table 2. The Theoretical Framework of Acceptability [67] was used in four of these reviews and defined acceptability as “a multi-faceted construct that reflects the extent to which people delivering or receiving a healthcare intervention consider it to be appropriate, based on anticipated or experiential cognitive and emotional responses to the intervention”. (p.8) This framework consists of seven constructs related to acceptability including affective attitude, burden, perceived effectiveness, ethicality, intervention coherence, opportunity costs and self-efficacy.

**Table 2:** Definitions, frameworks and commonly measured components in systematic reviews on acceptability of healthcare interventions (*n* = 132)

Frameworks (used in ***N*** = 6 reviews)	*N* (%)
• Asiimwe et al. (2012). Conceptual framework for exploring acceptance and use of mRDT Acceptance and use maybe be influenced by user attributes, the diagnostic tool and the health system. Attributes include learnability, willingness, suitability, satisfaction, efficacy and effectiveness.	1 (17)
• Sekhon et al. (2017) [67]. Theoretical Framework of Acceptability Acceptability is a multi-faceted construct that reflects the extent to which people delivering or receiving a healthcare intervention consider it to be appropriate. The Theoretical Framework of Acceptability includes affective attitude, burden, perceived effectiveness, ethicality, intervention coherence, opportunity costs, and self-efficacy	4 (66)
• Rosenstock et al. (1966). Health beliefs model An individual’s course of action depends on their perceptions of benefits and barriers including perceived susceptibility, perceived severity, perceived benefits, perceived barriers, cue to action and self-efficacy.	1 (17)
**Commonly measured components**	
• Attrition/dropout rates	44 (33)
• Perception of users including satisfaction, experience, views (receivers of interventions and those delivering)	40 (30)
• Adherence/compliance	17 (13)
• Adverse events/side-effects	4 (3)
• Recruitment	5 (4)
• Other (effectiveness, cost-effectiveness, efficacy, future intentions, likelihood to recommend to others or repeat intervention)	4 (3)

#### Fidelity

Of the 41 reviews exploring fidelity of a healthcare intervention, 35 included an *a priori* definition. Almost all reflected a dictionary definition of fidelity [68] with terms such as “integrity” and “delivered as intended” and “accuracy and consistency” included in their descriptions.

Thirty-six reviews measured four or more components as part of their assessment of fidelity of a healthcare intervention including adherence to the protocol (76%), dose delivered and received (76%) and provider training (49%). Thirty-four publications on fidelity of a healthcare intervention explicitly used a framework to guide their review, as described in Table 3. The framework used in 19 of these cases was from the National Institute of Health Behavior Change Consortium (NIHBCC) [69] which includes measures of study design, training, delivery of treatment, receipt of treatment and enactment of treatment. The NIHBCC describe fidelity as “the methodological strategies used to monitor and enhance the reliability and validity of behaviour interventions”. (p.443)

**Table 3:** Definitions, framework and commonly measured components in systematic reviews on fidelity of healthcare interventions (*n* = 41)

Frameworks (used in ***N*** = 34 reviews)	*N* (%)
• Dane & Schneider (1998) Fidelity of intervention should include a measure of adherence to the program, dose, quality of program delivery, participant responsiveness and program differentiation.	7 (21)
• Borrelli et al., (2005). National Institute of Health Behavioral Change Consortium Treatment fidelity should be assessed using 5 categories including design, training, delivery, receipt and enactment.	19 (56)
• Carroll et al., (2007) Implementation fidelity should include the measurement of adherence (content, frequency, duration and coverage) and moderators (intervention complexity, facilitation strategies, quality of delivery and participant responsiveness).	2 (6)
• Steckler & Linnan (2002) Public health interventions should be measured and evaluated against seven different components including context, reach, dose delivered, dose received, fidelity, implementation and recruitment.	2 (6)
• Moncher & Prinz (1991) Fidelity requires a clear definition of the treatment, training in delivery of the protocol, treatment manuals, supervision and adherence to the treatment protocol through treatment verification.	2 (6)
• Sidani & Sechrest (1999) Fidelity of implementation should include conceptualisation of the problem, operationalisation of the theory and specification of mediating processes and outcome variables.	1 (3)
• Perepletchikova, Treat & Kazdin (2007). Implementation of Treatment Integrity Procedures Scale (ITIPS) Evaluation of treatment integrity in psychotherapy research should include four domains: establishing, assessing, evaluating and reporting fidelity along with therapist treatment adherence and competence	1 (3)
**Commonly measured components**	
• Dosage	31 (76)
• Adherence/compliance	31 (76)
• Quality	9 (22)
• Responsiveness	31 (76)
• Training	20 (49)
• Other (program differentiation, supervision, treatment manual, environmental design, therapist qualifications, theory)	11 (27)

#### Feasibility

Thirteen of 65 reviews defined feasibility using terms such as “practicality” and “ease of delivery” and “possible to undertake”. Seven of these 13 papers noted the importance of context and broader system factors when considering the “possibility of what could be done”, such as physical space, ongoing funding and political support [39, 70–75].

The two most frequently measured constructs within the concept of feasibility were adherence to the study protocol (34%) (i.e. the same as the operationalisation of fidelity) and perceptions of key stakeholders (33%), including those providing and receiving the healthcare intervention (23%). There was a significant overlap between feasibility and acceptability with a number (22%) of feasibility reviews incorporating acceptability as a construct to be measured within feasibility.

Five reviews referred to a feasibility framework (Table 4) with the most commonly used being Bowen et al’s publication on designing feasibility studies [76]. This highly cited publication does not provide a definition of feasibility but does identify eight areas of focus that should be addressed in feasibility studies including acceptability, demand, implementation, practicality, adaptation, integration, expansion and limited efficacy testing. Bowen et al. define implementation as the extent to which the intervention can be delivered as planned which is synonymous with fidelity.

**Table 4:** Definitions, frameworks and commonly measured constructs in systematic reviews on feasibility of healthcare interventions (*n* = 65)

Frameworks (used in ***N*** = 5 reviews)	*N* (%)
• Bowen et al., (2009) Feasibility studies should address eight general areas including acceptability, demand, implementation, practicality, adaptation, integration, expansion and limited efficacy testing.	3 (60)
• Bird et al., (2014). The Structured Assessment of Feasibility (SAFE) Feasibility of complex interventions within mental health services are influenced by 16 factors such as staff training, intervention complexity, time, supervision and adverse events	1 (20)
• Joanna Briggs Institute Measure of feasibility, appropriateness, meaningfulness and effectiveness (FAME)	1 (20)
**Commonly measured components**	
• Dropouts/attrition	9 (14)
• Adherence/compliance	22 (34)
• Completion	11 (17)
• Recruitment	13 (20)
• Cost-benefit/economic feasibility	7 (11)
• Adverse events/side-effects	8 (12)
• Acceptability	10 (15)
• Perceptions of users (satisfaction, ease of use, perceived enjoyment)	15 (23)
• Other (including but not limited to context-specific and operational issues, intervention practicality/acceptability/integrity, training, equipment, time, knowledge, contraindications)	25 (38)

Almost a third (32%) of the reviews found the healthcare intervention to be feasible, with the remaining being a mixture of not feasible or unable to establish feasibility due to lack of information in the empirical studies.

#### Scalability

Healthcare interventions may be scaled up to different populations and/or settings. Eleven reviews explored scalability of a healthcare intervention with nine presenting an *a priori* definition including terms such as “deliberate efforts” and “expanding or increasing the impact”. Six of these definitions also included the need for a healthcare intervention to be proven effective prior to scaling up.

Five reviews measured four or more constructs with organisation, community and sociocultural factors being the most frequently reported measures (45%) followed by resources, economic viability (18%) and adaptation of the intervention (18%). Only one of the reviews definitively found that the healthcare intervention had been successfully expanded across different settings or populations [77]. The majority were unable to reach a conclusion due to lack of data in the included studies.

Four different scalability frameworks (Table 5) were described and used in four of the 11 reviews [59, 78–80]. These were the World Health Organisation ExpandNet Scaling-Up Framework [81], the Intervention Scalability Assessment Tool (ISAT) [82], the Assess, Innovate, Develop, Engage, Devolve (AIDED) model [83] and the Non-adoption, Abandonment, Scale-up, Spread, Sustainability (NASSS) framework [84]. Commonalities across the four frameworks include the intervention, the strategic/political context to support scale-up and resources to support and sustain the scale-up process. Scalability was defined in a similar way within these frameworks as the capacity or ease with which an intervention or innovation that had been proven effective could be expanded to other settings or populations [81, 82].

**Table 5:** Definition, frameworks and commonly measured constructs in systematic reviews on scalability of healthcare interventions (*n* = 11)

Frameworks (used in ***N*** = 4 reviews)	*N* (%)
• WHO/ExpandNet. Scaling-up framework Scaling up consists of five elements: the innovation, resource team, user organisation, broader environment and the scaling strategy	1 (25)
• Milat et al., (2020) [82]. Intervention scalability assessment tool (ISAT) Assessment of scalability includes five domains: the problem, the intervention, strategic/political context, evidence of effectiveness, intervention costs and benefits, fidelity and adaptation, reach and acceptability, delivery setting and workforce, implementation infrastructure and sustainability.	1 (25)
• Bradley et al., (2012) [83]. The AIDED model Scalability consists of five interrelated components: the landscape, innovation to fit user receptivity, support, engagement of user groups and effort for spreading innovation.	1 (25)
• Greenhalgh et al., (2017) [84]. NASSS Framework	1 (25)
**Commonly measured components**	
• Adaptation	2 (18)
• Resources	4 (36)
• Partnerships/collaborations	1 (9)
• Organisation/community/sociocultural factors	5 (45)
• Cost-benefit/economic feasibility	2 (18)

#### Sustainability

Of the 25 reviews on this concept, 15 included a definition with common use of terms such as “continuation” and “extended period of time”. Seven of these definitions also included the notion that sustainability is about the maintenance of the intervention or program after initial funding or implementation efforts have ceased [58, 85–90].

Constructs typically measured in reviews of sustainability of healthcare interventions included organisation- or community-specific factors (36%), continuation of the intervention beyond a specified period of time (24%), established collaborations or partnerships (12%) and resources (8%). Only one of the reviews [91] found that sustainability had been achieved for the healthcare intervention under investigation. A number of other reviews [92–94] were unable to draw a definitive conclusion due to inconsistent definitions and measures of sustainability within the empirical literature in the reviews.

Seven different sustainability frameworks (Table 6) were referred to in nine of the systematic reviews. The two most frequently used frameworks included ongoing maintenance of benefits from the intervention, capacity building and integration of the intervention or program within the organisation [95, 96]. Moore et al. developed a comprehensive definition of sustainability based on five constructs including continuation of a program or intervention of implementation strategies or individual behaviour change, after a defined period of time, with or without adaptations but continuing to produce benefits for the individual and/or systems [95].

**Table 6:** Definitions, frameworks and commonly measured constructs in systematic reviews on sustainability of healthcare interventions (*n* = 25)

Frameworks (used in ***N*** = 9 reviews)	*N* (%)
• Scheirer (2005) Sustainability should be measured across three levels for sustainability: individual, as continuing to deliver services that are beneficial; organisation, as maintaining the programme and community, as maintaining capacity.	1 (11)
• Cekan and Zivetz (2016) Sustainability should measure if the program has incorporated a theory of change, presence of explicit sustainability goals in a monitoring and evaluation plan, methods for identifying unexpected outcomes; funding, capacity development and collaboration	1 (11)
• Moore et al., (2017) [95] Five key sustainability constructs describe individual and organisational capacity based on time, behaviour, adaptation of the program/intervention, ongoing benefits and ongoing delivery of the program/intervention	2 (22)
• Shediac-Rizkallah & Bone (1998) [96] Sustainability can be measured through maintenance of health benefits, integration of the program within an organisation and community capacity building	2 (22)
• Greenhalgh et al., (2017) [84] NASSS Framework The framework consists of 7 domains (condition, technology, value proposition, adopter system, organisation, wider system and adaptation over time) and numerous subdomains.	1 (11)
• McLeroy et al., (1998) Health related behaviour is influenced by individual factors, interpersonal factors, organisation factors, community factors and public policy factors.	1 (11)
• Lennox, Maher & Reed (2018). Consolidated framework for sustainability constructs in healthcare. Includes 40 constructs across six domains: the organisational setting, negotiating initiative processes, resources, the external environment, the initiative design and delivery and the people involved.	1 (11)
**Commonly measured components**	
• Time (endurance of intervention/program beyond a period of time)	6 (24)
• Training	3 (12)
• Resources	2 (8)
• Partnerships/collaborations	3 (12)
• Organisational/community factors	9 (36)

In summary, two key findings emerged from the overview of reviews. First, the current literature suggests that the concepts are related, although there was some variation in the terms used. For example, reviews on feasibility of a healthcare intervention measured ‘implementation’ of the intervention, defined as the extent to which the intervention can be delivered as planned, also known as fidelity. Similarly, reviews on sustainability, also known as maintenance, measured resources, funding and organisational factors, which were all measures frequently included within reviews on feasibility of a healthcare intervention. The second key finding is that although acceptability appeared to be an important factor contributing to fidelity, additional factors were required, for example provider training. Similarly, although fidelity was an important factor contributing to feasibility, additional factors were required, for example funding and other resources such as physical space.

### Step 2: Modified Nominal Group Technique

The first group meeting took place online using ZOOM [97] in March 2021. Three themes were identified from the group discussion, as follows:

Theme 1: It is plausible that the concepts influence implementability of a healthcare intervention.

Following presentation of the findings of the overview of reviews on each concept, participants agreed the theoretical plausibility of a framework of implementability for healthcare interventions that includes all five concepts. This was further consolidated through sharing their own real-world research and clinical experiences as described in Tables 7, 8, 9, 10 and 11.

**Table 7:** Scenario illustrating influence of acceptability on implementability

**Study title** Does prospective acceptability of an intervention influence refusal to participate in a randomised controlled trial? **Background** Blepharospasm and hemifacial spasm are currently managed by regular Botox injections, given approximately every 2 months (appointments scheduled by the physician). But the timeline to return of symptoms following treatments is variable. It is possible that patient-initiated appointments (i.e. when symptoms flare up) could result in a more efficient and effective service. This possibility was tested in a randomised trial. A qualitative investigation of acceptability was conducted as a sub-study. **Aims** To apply the Theoretical Framework of Acceptability (TFA; consisting of 7 component constructs) to explore: (1) patient-reported reasons for declining to participate in the trial; and (2) associations between decliners’ perceptions of acceptability and their non-participation. **Method** Eligible patients (n = 242) were approached to participate in the trial. Phase 1: decliners provided a brief reason for refusal. We analysed the reasons descriptively and reviewed them against the TFA constructs. Phase 2: We invited consecutive decliners to participate in short semi-structured interviews, to explore their reasons for refusal in more depth. Interviews were transcribed and analysed, with the TFA as a coding framework. **Results** Eighty-seven (36%) eligible patients refused trial participation; all provided a reason. From interviews with 15 decliners, four key beliefs about acceptability were identified: happy with standard care, anticipated burden of the patient-initiated service, lack of confidence in ability to engage with new service and uncertainties about the effectiveness of new service. Two themes reflected non-TFA factors: trial participation was a low priority and the burden of completing trial documentation. **Conclusion** Reasons for refusing trial participation were often, but not always, associated with intervention acceptability. **Relationship to implementability** 1. Three factors could be improved to enhance acceptability of the new service: reducing burden, enhancing patient support to increase confidence (making sure they are able to make contact by phone), and ensuring that the new service is perceived to be workable (increasing available appointment spaces). 2. Unless these factors can be satisfactorily addressed it appears that the new service would not be implementable. **Reference** Sekhon, M., Cartwright, M., Lawes-Wickwar, S., et al. (2021). Does prospective acceptability of an intervention influence refusal to participate in a randomised controlled trial? An interview study. *Contemporary Clinical Trials Communications*, 21, 100698.

**Table 8:** Scenario illustrating influence of fidelity on implementability

**Study title** Fidelity of an allied health prehabilitation service for haematologic patients receiving high dose chemotherapy in a large cancer centre. **Background** Cancer prehabilitation can reduce post-treatment complications, enhance functional capacity, and empower patients to withstand treatment stressors. As part of a larger study, we evaluated the fidelity of a multidisciplinary allied health (exercise, nutrition, and psychology) prehabilitation clinical service as part of routine care in haematologic cancer patients receiving intensive conditioning chemotherapy prior to an autologous stem cell transplant (AuSCT). **Method** We retrospectively analysed data routinely collected from patients referred between March 2019 and March 2020. All patients considered for AuSCT at a tertiary specialist cancer centre were eligible to participate. The prehabilitation intervention included individualised exercise prescription and input from other allied health teams. Fidelity of the prescribed exercise program was assessed along the pathway from referral to the AuSCT service through to receipt by patients. **Results** 183 patients were referred to the AuSCT service, 133 (73%) were referred into the prehabilitation service, 128 (96%) were eligible and 116 (91%) participated. Fidelity of exercise prescription was moderate with 72% of patients receiving the intended aerobic and resistance exercise intervention. Hence, 83 (65%) of the original 128 eligible patients actually received the exercise component of the intervention. **Conclusion** Although the prehabilitation service was well adopted by clinicians, there was some room for improvement in terms of the objective of providing all eligible patients with exercise prehabilitation support. **Relationship to implementability** 1. Only two-thirds of eligible patients received the intervention as intended. 2. Although the intervention appears to be implementable, further support is needed to increase consistency and equity of delivery. **Reference** Crowe, J., Francis, J. J., Edbrooke, L., et al. Impact of an allied health prehabilitation service for haematologic patients receiving high dose chemotherapy in a large cancer centre. Under review.

**Table 9:** Scenario illustrating influence of feasibility on implementability

**Study title** Feasibility of conducting family meetings for hospitalised palliative care patients. **Background** A family meeting is a clinical tool for healthcare providers to facilitate communication with patients with advanced disease and their family caregivers. Despite family meetings being advocated as standard practice, minimal evidence existed regarding the balance between costs and benefits. The economic feasibility (healthcare utilisation) of providing a structured family meeting for hospitalised palliative care patients was evaluated as part of a larger cluster randomised trial. **Method** A pragmatic cluster randomised control trial was conducted across three major Australian hospitals. Patients admitted or referred to specialise palliative care units, and their primary family caregiver, were invited to participate. The intervention consisted of a single structured family meeting tailored according to the individual needs of the participant, family caregiver and treating team. The control group received usual care. Caregiver psychological distress, patient outcomes and healthcare utilisation data were compared between the two groups. **Results** A total of 297 dyads were recruited and randomised: control group (*n* = 153); intervention group (*n* = 144). The intervention group demonstrated significantly lower psychological distress (Diff: − 1.68, *p* < 0.01) and higher preparedness (Diff: 3.48, *p* = 0.001) at Time 2. No differences were identified for quality of end-of-life care or health resource utilisation. **Conclusion** Family meetings may assist in reducing family caregiver distress and preparing individuals for their caregiving role. The results also suggest that family meetings do not increase health service utilisation costs; however, this aspect of feasibility requires further examination. **Relationship to implementability** 1. Routinely conducting family meetings may not incur additional demands on health care utilisation. 2. Family meetings appear to be implementable but further investigation of other feasibility factors is required. **Reference** Hudson, P., Girgis, A., Thomas, K., et al. (2021). Do family meetings for hospitalised palliative care patients improve outcomes and reduce health care costs? A cluster randomised trial. *Palliative Medicine*, 35(1), 188–199.

**Table 10:** Scenario illustrating influence of fidelity and feasibility on sustainability of a healthcare intervention

**Study title** Factors affecting sustainability of a quality improvement policy on medications while fasting for surgery. **Background** Several adverse events associated with patients missing medications while fasting for surgery led to a quality improvement project that aimed to simplify and standardise oral restriction terminology and medication administration instructions to reduce confusion and unwanted practice variations when patients had oral intake restrictions such as fasting for surgery. **Method** A companion qualitative study to this quality improvement program was conducted after the roll out of the intervention: a new policy about medications and restrictions in oral intake. **Results** Before the quality improvement intervention, there was confusion, lack of clarity and guidance, and lack of experience and confidence in managing medications when patients had oral restrictions. After the rollout, there was improved clarity and decision support; but problems included lack of awareness about the policy, particularly due to staff movement and turnover; and individual interpretation and acceptance of the policy. Sustainability of the project appears dependent on continuing the role of a project officer combined with educators. These roles also appear important for scaling up the program within one hospital and essential for implementability elements of scaling up, acceptability, and fidelity in other hospitals. **Conclusion** Elements needed for greater sustainability included strategies and resources to 1) educate staff; 2) minimise variation, and optimise fidelity, in interpreting information; and 3) deal with continuous staff changes. **Relationship to implementability** 1. Routinely conducting family meetings may not incur additional demands on health care utilisation. 2. The elements of implementability appeared to interact and cannot be viewed insolation. 3. Sustainability was heavily affected by staff changes and requires ongoing investment. **Reference** To, T‐P, Dunnachie, G, Brien, J‐a, Story, DA. Surgical nurses' perceptions and experiences of a medications and oral restrictions policy change: A focus group study. J Clin Nurs. 2019; 28: 3242– 3251.

**Table 11:** Hypothetical scenario illustrating influence of acceptability, fidelity and feasibility on scalability of a health intervention

**Scenario title** Scalability of a digital health intervention **Background** A suite of new care models was designed and deployed at a hospital to manage the COVID-19 pandemic. One model supported patients in monitoring symptoms at home and advised patients about when and if they needed medical care and could present to the hospital. This virtual care model leverages technology to connect the patient with best evidence and provide targeted advice from their provider when needed. Outcomes included avoidance of emergency room and hospital overcrowding, lower cost to the patient, increase patient control and peace of mind. Many other conditions could be managed with a virtual care model and, after success of the COVID-19 model, the hospital would like to scale the model to better provide care for older adults with complex comorbidities. They decide to adapt the model to patients with chronic respiratory disease, who are one of the main sources of unnecessary Emergency Department admissions.	
**Barriers to scalability**	**Scenario**
Acceptability	Initial deployment used a homegrown database with a website link • Older adults often have lower digital literacy • Many older adults do not have daily access to a computer
Fidelity	The intervention was deployed with urban patients, highly educated, and good internet connection • The deployment will be different with rural patients with poor internet
Feasibility	Will require primary care integration. • Dispersed, independent GP clinics make it difficult to disseminate • There is no reimbursement model for GPs to look at panels of patients • Cannot currently exchange information between hospital EMR and many different primary care electronic medical records Captures oxygen saturation with a digital device • Emergency funding during pandemic not available for continuing program • Device does not work as well on patients with darker skin shades Enrolling patients relied on their coming to ED with COVID-19 symptoms • Identifying eligible patients will require new technology • All data required for eligibility requirements may not be digital or may not be sensitive/specific enough
**Conclusion** Adapting a digital health intervention to a new population or setting can be like starting over again due to differences in IT infrastructure, digital literacy, and funding models **Relationship to implementability** Scalability of a digital intervention like this scenario encompasses acceptability, fidelity, and feasibility	

Theme 2: The concepts appear to be related to one another.

Participants were asked to consider the question “in your view, is it plausible that the concepts are related to each other?” All agreed that it was possible that the concepts were interdependent and should be considered together when developing and implementing a healthcare intervention. They suggested that individual concepts were necessary but insufficient on their own to ensure implementability of a healthcare intervention. Participants also identified that in many of the clinical scenarios they shared, often more than one concept was involved in the final outcome. For example, it was identified that the hypothetical scalability scenario included issues with feasibility of a device, acceptability of an online system and lack of procedural information relevant for fidelity.

Theme 3: The preliminary implementability framework could be amended to better represent the relationships between the concepts.

Although all participants agreed that the concepts were related, they were not in agreement that the preliminary framework adequately represented the nature of their interdependence. Some participants felt more detail was needed to explain how the concepts related whilst other participants felt that some concepts required greater representation than others. All participants were asked to further consider the framework over the following week and provide feedback and revisions.

### Step 3: Revising the framework

**Fig. 4:**
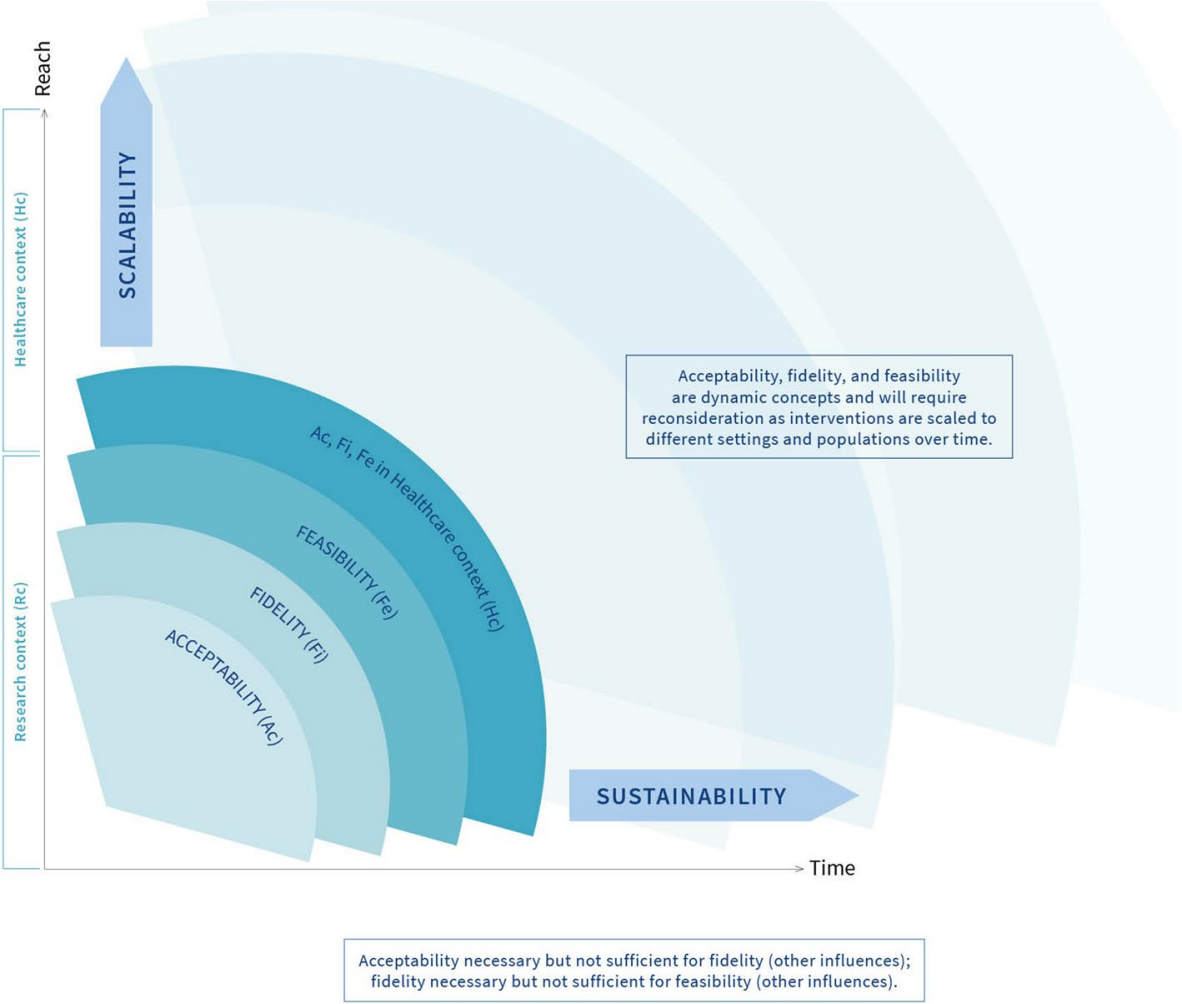
Conceptual framework of implementability of healthcare interventions

Following the first group discussion and feedback from the participants, the preliminary framework was revised, resulting in three options presented to consensus panel participants as three figures. These three draft frameworks included the revisions requested by the participants such as annotations on the first framework for option one, different graphics representing different relationships between the concepts on the second option and a combination of options one and two for the third framework option. Participants were asked to consider the three options and silently vote on their preferred framework and provide any further feedback via return email. The feedback was considered and integrated by two authors (MK and JF) resulting in a further version of the framework (Fig. 4). All participants agreed this final version.

The framework as depicted in Fig. 4 is designed to guide research activities, chronologically and iteratively, from left to right and from bottom to top. Commencing in the research context, it is proposed that acceptability is the first concept to assess, during intervention development and work-up of supporting documentation and resources (intervention protocol, training manual, patient information leaflet, data and technology requirements, validation of digital components, etc.). If acceptability is adequate to providers and potential recipients, it is appropriate to deliver the intervention to assess fidelity as delivered and as received. Without adequate acceptability, providers and recipients are unlikely to engage with the intervention, and hence, fidelity will be low. Adequate fidelity will also require other enabling factors such as provider training and confirmed information flow. Without adequate fidelity, it would be wasteful to conduct a feasibility study. Factors such as appropriate resources, workforce, technology, and management will be required for feasibility. If feasibility (supported by acceptability and fidelity) in the research context is adequate, it is appropriate to consider testing acceptability, fidelity and feasibility in the healthcare context. If adopted consistently in one healthcare context, it is appropriate to consider scaling the intervention to other settings, provider groups and patient groups. In each new setting, it would be wise to re-assess acceptability, fidelity and feasibility as described above because adequate feasibility in one setting at one time, whilst a positive sign, is not a guarantee that the intervention will be feasible in other settings. Similarly, over time, the factors that support feasibility may change, thus threatening sustainability. It would therefore be prudent to continue to assess the factors affecting feasibility over time to detect any problems that need to be addressed.

## Discussion

Based on the findings from the overview of reviews and the group consensus process, we propose a framework of implementability of healthcare interventions which includes five key concepts, namely, acceptability, fidelity, feasibility, scalability and sustainability. The framework illustrates the interrelationship between the concepts and chronology, with acceptability, fidelity and feasibility requiring investigation during early stages of the development of a healthcare implementation, including during proof-of-principle studies and pragmatic evaluations of intervention effectiveness at one point in time and in one specific context. Acceptability is a necessary but not sufficient condition for fidelity, and similarly, fidelity is a necessary but not sufficient condition for feasibility. All three concepts are context- and population-dependent and will require reinvestigation as the healthcare intervention is scaled to different settings and populations and over time. We argue that there is an association between the concepts, with acceptability, fidelity and feasibility influencing the scalability and sustainability of a healthcare intervention. We are not suggesting that there is a causal relationship between the concepts, rather, scalability and sustainability depend on the pre-conditions acceptability, fidelity and feasibility of the healthcare intervention, and these concepts should be re-examined over time, and as the healthcare intervention is implemented with different populations or in different settings. This argument is consistent with other frameworks on scalability and sustainability, including the Intervention Scalability Assessment Tool [82] and the Dynamic Sustainability Framework [98], both of which suggest that feasibility, acceptability and fidelity must be considered in the planning for scaling up and sustainability of a healthcare intervention. The Consolidated Framework for Implementation Research [12] suggests that the outer setting, including the social and economic context, can influence implementation. Our proposed framework encourages the researcher to prospectively assess acceptability, fidelity and feasibility in both the inner and outer contexts as the key stakeholders are likely to be different.

From the 252 reviews identified in the overview, the majority did not provide a definition of the concept. Rather, the reviews used measurement approaches which implied a definition. For example, feasibility was typically defined by measuring components such as compliance to the intervention, dropouts, recruitment rates and adverse events. There was conflating of terms, with feasibility and safety used interchangeably in several reviews, particularly drug feasibility reviews. Although frameworks were identified for all five concepts, they were not frequently used in the reviews identified in the overview. Most of the frameworks included conceptual definitions and operationalisation of the concept, but these varied between frameworks. It is difficult to test the influence of these concepts on the implementability of a healthcare intervention without consistent definitions, descriptions, operationalisation and measurement approaches for these concepts.

Of all the concepts explored in the systematic overview, scalability and sustainability of healthcare interventions were not often achieved. These findings suggest that healthcare interventions may be found to be effective, acceptable and feasible in the development or pilot phase, but this does not guarantee successful scale-up or sustainment of the intervention over time. We propose that the framework of implementability can provide a dynamic, longitudinal perspective of intervention development where researchers consider acceptability, fidelity and feasibility during the earlier phases of intervention development and implementation, and iteratively re-evaluate these factors as the healthcare intervention is scaled to different settings and over time.

Other rigorous frameworks, such as RE-AIM and the Implementation Outcomes Framework, propose that sustainable adoption and implementation of healthcare interventions require consideration of many like concepts such as acceptability, reach, effectiveness, adoption, implementation and maintenance [99, 100]. Whilst there are some similarities in the concepts contained in these frameworks and our proposed framework of implementability, the latter is explicitly concerned with the prospective and ongoing identification of factors that will influence scalability and sustainability of a healthcare intervention. It should also be noted that prospective identification of factors that influence implementability of healthcare interventions is receiving growing attention in the literature, particularly in relation to reducing avoidable research waste [101]. A recent publication developed a framework to assist researchers to prospectively cost the different phases of healthcare interventions. The authors argue that implementation costs are often underestimated or not included in cost-effectiveness analyses. This in turn contributes to research waste which may have been avoided through a more systematic and earlier approach to identifying the factors that support the translation of effective interventions into real-world settings, and prospectively costing the implementation of these.

It has been argued that involving end-users in the development and implementation of healthcare interventions may improve outcomes through enhanced relevance, acceptability and feasibility [102]. We propose that our framework of implementability could be used to test these assumptions. We recommend that researchers prospectively set criteria to inform the decision about whether to abandon, amend or proceed with the intervention, depending on the outcomes of the feasibility study [103]. Table 12 illustrates how the framework of implementability could be used to prospectively guide the ongoing implementation activities of healthcare interventions. Enabling factors were identified from published frameworks and reviews included in step 1, though this is not an exhaustive list and is likely to be influenced by context. For example, scalability of healthcare interventions to low- and middle-income countries may be more, or less, enabled by factors that differ from those in high-income countries [104]. The framework of implementability of healthcare interventions may be particularly helpful in guiding effectiveness-implementation hybrid designs, which aim to simultaneously evaluate effectiveness of the intervention in the real-world context and the implementation strategy [105–107].

**Table 12:** Example plan for using the framework of implementability

Concept	During development and early evaluation	After initial evidence of effectiveness	Enabling factors to consider
Acceptability	✓	✓	Intervention information/knowledge, experience of delivering or receiving the intervention
Fidelity	✓	✓	Acceptability, training, supervision, treatment manual
Feasibility	✓	✓	Fidelity, training, resources (equipment, physical space, time), social/organisational/political support
The factors above to be investigated iteratively with stakeholders in the inner setting The factors below to be investigated iteratively with a broader range of stakeholders (inner and outer settings)			
Scalability	✕	✓	Acceptability, fidelity, feasibility, training, resources (equipment, physical space, time), social/organisational/political support, partnerships and collaborations
Sustainability	✕	✓	Acceptability, fidelity, feasibility, training, resources (equipment, physical space, time), social/organisational/political support, partnerships and collaborations

We propose that empirical investigation of the framework of implementability is required to answer the following questions:

What is the nature of the relationship between the key concepts? (e.g. linear, curvilinear or threshold?)

Do acceptability, fidelity and feasibility predict scalability and sustainability as proposed in the framework?

Can identified deficits in constructs of the framework be addressed to enhance the implementability of effective interventions?

## Strengths and limitations

To our knowledge, this is the first review that considers all five key concepts in published reviews on healthcare interventions. The overview collated and described important information on concepts that are increasingly being assumed to influence implementation of healthcare interventions. The development of a framework utilising well-established consensus methods is another strength. Although one author was responsible for most of the screening, data extraction and coding, independent extraction by a second author of 10% of the reviews confirmed reliability of the extraction process.

In order to the make the overview of reviews feasible, we only focused on publications that had one or more of the concepts in the title and/or abstract. Therefore, it is possible we may not have identified some reviews that were relevant. It must also be noted that the framework of implementability of healthcare interventions is untested. We propose that it articulates some of the untested assumptions in the current literature on implementation science and have suggested some approaches for empirical evaluation of the framework.

We do not propose that interventions with high implementability will automatically result in high uptake. As we argued in the background to this paper, the features of interventions may interact with top-down and bottom-up implementation activities, and with contextual factors, to achieve consistent uptake into routine practice. Our argument is that these implementation activities are more likely to be effective if implementability of the intervention is high.

## Conclusions

The framework developed in this study can inform research that aims to prospectively and iteratively identify the likely implementability of evidence-based healthcare interventions. We suggest that the framework be tested empirically through studies that examine the actual uptake of interventions, across settings and over time, compared with prospective assessments of the independent variables (acceptability, fidelity and feasibility) and the outcome variables (scalability and sustainability) in the framework. We recommend that, to avoid research waste, implementability should be assessed, and enhancements made, during the clinical evaluation stages of the development of interventions [1, 3]. This would potentially accelerate their uptake into clinical practice.

## Supplementary Information

**Additional file 1.** Search strategy.

**Additional file 2.** Papers included in the overview.

**Additional file 3.** Characteristics of studies included in the overview of reviews (n=252).

## Data Availability

All data generated during this study are included either within the text or as an additional file.

## Abbreviations

AIDED: Assess, Innovate, Develop, Engage and Devolve
CFIR: Consolidated Framework of Implementation Research
FAME: Feasibility, Appropriateness, Meaningfulness and Effectiveness
IOF: Implementation Outcomes Framework
ISAT: Intervention Scalability Assessment Tool
ITIPS: Implementation of Treatment Integrity Procedures Scale
NASSS: Non-adoption, Abandonment, Scale-up, Spread and Sustainability
NGT: Nominal Group Technique
NIHBCC: National Institute of Health Behavior Change Consortium
PRISMA: Preferred Reporting Items for Systematic Reviews
RE-AIM: Reach, Effectiveness, Adoption, Implementation and Maintenance
SAFE: Structured Assessment of Feasibility
SR: Systematic review
WHO: World Health Organisation
