# Supplementary material for: Implementability of healthcare interventions: an overview of reviews and development of a conceptual framework
Source: Implement Sci. 2022 Jan 27;17:10. doi: 10.1186/s13012-021-01171-7 (PMC8793098; doi:10.1186/s13012-021-01171-7)
Supplement: Supplementary file 3 — Additional file 3. Characteristics of studies included in the overview of reviews (n=252). [file 13012_2021_1171_MOESM3_ESM.docx]

Table 2 Characteristics of studies included in the overview of reviews (n=252)

| **ACCEPTABILITY** | | | | | | |
| --- | --- | --- | --- | --- | --- | --- |
| **Citation details** | **Type of review** | **Healthcare intervention** | **Number of studies included** | **Total number of participants** | **Language or country exclusions** | **Setting exclusions** |
| (Dai & Livesley, 2018) | Mixed methods | Preoperative psychological preparation programmes to reduce paediatric preoperative anxiety in elective surgery | 29 | 2,111 | Not available in English or Chinese | Not in hospital or home |
| (Scope et al., 2017) | Qualitative | Art therapy for people with non-psychotic mental health disorders | 12 | 199 | None reported | Not primary or secondary care |
| (Gallo et al., 2012) | Mixed methods | Female condom for dual protection | 10 | Not reported | None reported | None reported |
| (M. Berlim et al., 2013) | Systematic review | Bilateral repetitive transcranial magnetic stimulation for treating major depression | 7 | 279 | Not available in English | None reported |
| (Davis et al., 2014) | Mixed methods | Remote monitoring technology | 15 | Not reported | Not available in English | Not primary care |
| (Muftin & Thompson, 2013) | Mixed methods | Self-help for disfigurement | 11 | 666 | None reported | Not clinical or community |
| (Cleeve et al., 2019) | Systematic review | Medical management of intrauterine fetal death at 14-28 weeks of gestations | 16 | 1,890 | None reported | Not reported |
| (Forbes et al., 2019) | Mixed methods | Online supportive care interventions targeting men with a history of prostate cancer | 16 | 2,446 | Not available in English | n/a |
| (Aparício et al., 2016) | Systematic review | Transcranial direct current stimulation treatment in neuropsychiatry trials | 64 | 2,262 | Not available in English | Not reported |
| (Simmonds-Buckley et al., 2020) | Systematic review | NHS recommended e-therapies for depression, anxiety, and stress | 24 | 7,075 | Not available in English | Clinical  Community |
| (Simmonds-Buckley et al., 2019) | Systematic review | Group behavioral activation for depression among adults | 19 | Not reported | No language restrictions | No limit on settings |
| (Taleghani et al., 2019) | Systematic review | Partner notification for curable sexual transmitted infections in sub-Saharan Africa | 19 | Not reported | Not available in English  Not sub-Saharan Africa | None reported |
| (Pham et al., 2016) | Systematic review | Point-of-care CD4 testing on HIV continuum of care in low- and middle-income countries | 12 | Not reported | Not available in English  LMICs | Not in field |
| (Skea et al., 2019) | Qualitative review | Weight management programmes for adults with severe obesity | 33 | 644 + 153 | None reported | None reported |
| (Giles et al., 2015) | Mixed methods | Financial incentives for encouraging uptake of healthy behaviours | 81 | Not reported | Not available in English | None reported |
| (Hoskins et al., 2019) | Mixed methods | Financial incentives for health-related behavior change | 47 | Not reported | Not available in English | None reported |
| (Harichund & Moshabela, 2018) | Scoping review | HIV self-testing in sub-Saharan Africa | 11 | Not reported | Not available in English  Not sub-Saharan Africa | None reported |
| (Krause et al., 2013) | Mixed methods | HIV self-testing | 11 | Not reported | None reported | None reported |
| (Leistikow & Cervia, 2020) | Systematic review | HIV self-testing for adolescents and young adults by delivery model | 69 | Not reported | Not available in English | None reported |
| (Buckingham et al., 2021) | Mixed methods | Immediate postpartum and post-abortion long-acting reversible contraception provision to adolescents | 10 | Not reported | Not available in English | No exclusions |
| (Simon et al., 2019) | Mixed methods | Internet-based cognitive behavioural therapy (i-CBT) for post-traumatic stress disorder | 10 | 720 | Not available in English | Not online or mobile phone or remote |
| (Berry et al., 2016) | Systematic review | Interventions delivered online and through mobile phones for people who experience severe mental health problems | 49 | Not reported | Not available in English | Not online or mobile phone |
| (Westercamp & Bailey, 2007) | Systematic review | Male circumcision for prevention of HIV/AIDS in Sub-Saharan Africa | 13 | Not reported | Not available in English  Not sub-Saharan Africa | None reported |
| (Bautista et al., 2019) | Systematic review | Mindfulness-based interventions for substance use disorder | 17 | Not reported | Not available in English | None reported |
| (Sotirova et al., 2021) | Mixed methods | Online exercise-based interventions after breast cancer surgery | 11 | 965 | Not available in English | Not online |
| (Qiu et al., 2019) | Mixed methods | Psychosocial interventions for dementia caregivers | 42 | Not reported | Not available in English | None reported |
| (Liptrott et al., 2018) | Mixed methods | Telephone support as perceived by patients with cancer | 48 | Not reported | Not available in English | Not telephone |
| (Peters et al., 2014) | Mixed methods | Female condom by Sub-Saharan African women | 15 | Not reported | Not available in English  Not sub-Saharan Africa | None reported |
| (Marrazzo & Scholes, 2008) | Mixed methods | Urine-based screening for chlamydia trachomatis to asymptomatic young men | 21 | Not reported | None reported | Not non-STD clinic or home |
| (Morrell et al., 2016) | Systematic review | Interventions to prevent postnatal depression | 122 + 56 | Not reported | Not available in English | None reported |
| (Morgan et al., 2019) | Mixed methods | Self-sampling versus clinician-collected samples for HPV DNA testing | 23 | Not reported | None reported | None reported |
| (Marshall et al., 2019) | Mixed methods | HPV self-sampling among immigrant minority women | 28 | Not reported | Not available in English | None reported |
| (Babiano-Espinosa et al., 2019) | Systematic review | Internet cognitive behavioral therapy (iCBT)  for pediatric obsessive-compulsive disorder | 6 | 96 | Not available in English | No exclusions |
| (Driedger et al., 2018) | Qualitative | Infectious disease interventions among migrants in the EU/EEA | 11 | Not reported | Not available in English | None reported |
| (Faulkner et al., 2020) | Mixed methods | Light therapies to improve sleep in intrinsic circadian rhythm sleep disorders and neuropsychiatric illness | 77 | 2,872 | None reported | Not home or hospital or clinic or laboratory |
| (de Barros & Cardoso, 2016) | Mixed methods | Home fortification with vitamins and minerals in children aged 6 to 23 months | 17 | Not reported | Not available in English, Portuguese or Spanish | None reported |
| (Pellowski et al., 2016) | Mixed methods | Advancing partner notification through electronic communication technology | 23 | Not reported | None reported | None reported |
| (Figueroa et al., 2015) | Mixed methods | HIV self-testing among key populations | 23 | Not reported | None reported | None reported |
| (Strauss et al., 2015) | Mixed methods | Mindfulness-based interventions for people distressed by hearing voices | 15 | 479 | Not available in English | None reported |
| (M. T. Berlim et al., 2013) | Systematic review | Low frequency repetitive transcranial magnetic stimulation (rTMS) for treating primary major depression | 8 | 263 | Not available in English | None reported |
| (Chu et al., 2021) | Systematic review | Non-invasive brain stimulation on Alzheimer’s disease and mild cognitive impairment | 27 | 1,070 | No exclusions | No exclusions |
| (Morris et al., 2018) | Mixed methods | Communication training interventions for family and professional carers of people living with dementia | 38 | Not reported | Not available in English | None reported |
| (Odesanmi et al., 2013) | Systematic review | Home-based and clinic-based sampling methods for sexually transmissible infections screening in females aged 14-50 | 6 | 5,475 | Not available in English | Not home or clinic |
| (Ngo et al., 2011) | Systematic review | Medical abortion at home and in a clinic | 9 | 4,522 | None reported | Not home or clinic |
| (Zhou et al., 2020) | Systematic review | Antidepressants, psychotherapies, and their combination for acute treatment of children and adolescents with depressive disorder | 71 | 9,510 | No restriction on language or country | None reported |
| (Yuan et al., 2018) | Systematic review | Bibliotherapy for depression and anxiety disorders in children and adolescents | 8 | 979 | No restrictions on language | None reported |
| (Gerger et al., 2021) | Systematic review | Expressive writing treatments for adult trauma survivors | 44 | 7,724 | No restrictions on language | None reported |
| (Mutz et al., 2019) | Systematic review | Non-surgical brain stimulation for the acute treatment of major depressive episodes in adults | 113 | 6,750 | Not available in English | None reported |
| (Merz et al., 2019) | Systematic review | Pharmacological, psychotherapeutic, and combination treatments in adults with posttraumatic stress disorder | 12 | 922 | No restrictions on language | None reported |
| (Solmi et al., 2021) | Systematic review | Psychological interventions for the treatment of adult outpatients with anorexia nervosa | 16 | 1,047 | No restrictions on language | None reported |
| (De Crescenzo et al., 2018) | Systematic review | Psychosocial interventions for individuals with cocaine and amphetamine addition | 50 | 6,942 | No restrictions on language | None reported |
| (Zhou et al., 2015) | Systematic review | Psychotherapies for depression in children and adolescents | 52 | 3,805 | No restrictions on language | None reported |
| (Huang et al., 2020) | Systematic review | Treatments for restless legs syndrome in end-stage renal disease | 12 | 498 | No restrictions on language | None reported |
| (Craig et al., 2020) | Mixed methods | Compassion focused therapy | 29 | Not reported | Not available in English | None reported |
| (Andrews et al., 2018) | Systematic review | Computer therapy for the anxiety and depression disorders | 64 | 8,279 | Not available in English | None reported |
| (Pierret et al., 2020) | Mixed methods | Education and training interventions and support tools for school staff to adequately respond to young people who disclose self-harm | 8 | 1,058 | Not available in English | Not primary or secondary educational settings |
| (Lee et al., 2020) | Systematic review | Mindfulness-based stress reduction program on patients with elevated blood pressure or hypertension | 12 | 872 | Not available in Chinese or English | None reported |
| (Cuijpers et al., 2019) | Systematic review | Cognitive behavior therapy delivery formats in adults with depressions | 151 | 15,191 | None reported | None reported |
| (Taylor et al., 2018) | Narrative review | Continuous glucose monitoring for type 2 diabetes management | 11 | 5,542 | Not available in English | None reported |
| (Berlim et al., 2014) | Systematic review | Deep brain stimulation (DBS) of the subgenual cingulate cortex for treatment-resistant depression | 4 | 66 | Not available in English | None reported |
| (Cremers et al., 2019) | Mixed methods | Low-intensity psychological interventions on the well-being of older adults | 26 | Not reported | None reported | None reported |
| (Robinson et al., 2007) | Mixed methods | Non-pharmacological interventions to reduce wandering in dementia | 11 | Not reported | No restrictions on language | No exclusions |
| (Coorey et al., 2018) | Mixed methods | Mobile applications for cardiovascular disease self-management | 10 | 607 | No restrictions on language | Not home |
| (Gilbey et al., 2020) | Mixed methods | Digital health interventions for LGBTIQ+ young people | 38 | Not reported | Not available in English | None reported |
| (Reilly et al., 2016) | Mixed methods | Chronic kidney disease management programs for indigenous people in Australia, New Zealand and Canada | 10 | Not reported | Not available in English  Not Australia, New Zealand or Canada | None reported |
| (Sjöström et al., 2017) | Mixed methods | First-trimester medical termination of pregnancy performed by non-doctor providers | 6 | Not reported | No restrictions on language | None reported |
| (Kriston et al., 2014) | Systematic review | Acute treatments for persistent depressive disorder | 56 | 5,806 + 5,348 | None reported | None reported |
| (Yang et al., 2017) | Meta-analysis | Cognitive behavioral therapy for depression in children | 9 | 306 | No restrictions on language | None reported |
| (Chou et al., 2021) | Network meta-analysis | Different interventions for acrophobia | 17 | 946 | No restrictions on language | None reported |
| (Okumura & Ichikura, 2014) | Network meta-analysis | Group cognitive behavioral therapy for depression | 35 | Not reported | Not available in English | None reported |
| (Pu et al., 2017) | Meta-analysis | Interpersonal psychotherapy for depression in adolescents | 7 | 538 | No restrictions on language | None reported |
| (Davies et al., 2018) | Network meta-analysis | Interventions for attenuated positive psychotic symptoms in individuals at clinical high risk of psychosis | 14 | 1,707 | No restrictions on language | None reported |
| (Goldberg et al., 2020) | Meta-analysis | Mindfulness based interventions for military veterans | 20 | 898 | No restrictions on language | None reported |
| (Mutz et al., 2018) | Meta-analysis | Non-invasive brain stimulation for the treatment of adult unipolar and bipolar depression | 56 | 3,058 | Not available in English | None reported |
| (Yin et al., 2021) | Meta-analysis | Parent-only group cognitive behavioral intervention for treatment of anxiety disorder in children and adolescents | 6 | 407 | No restrictions on language | None reported |
| (Yang et al., 2019) | Meta-analysis | Psychological interventions for social anxiety disorder in children and adolescents | 17 | 1,134 | No restrictions on language | None reported |
| (Turrini et al., 2019) | Meta-analysis | Psychosocial interventions in asylum seekers and refugees | 26 | 1,959 | No restrictions on language | None reported |
| (Zhang et al., 2017) | Meta-analysis | Psychotherapy for anxious young children | 7 | 358 | No restrictions on language | No exclusions |
| (Moffa et al., 2020) | Meta-analysis | Transcranial direct current stimulation (tDCS) for major depressive disorder | 9 | 572 | No restrictions on language | None reported |
| (Rehackova et al., 2016) | Meta-analysis | Very low energy diets in overweight and obese people with type 2 disabetes mellitus | 9 | 346 | No restrictions on language | None reported |
| (Bernardy et al., 2018) | Meta-analysis | Cognitive behavioural therapies in fibromyalgia syndrome | 296 | 1,236 + 1,246 | No restrictions on language | None reported |
| (Samara et al., 2020) | Network meta-analysis | Available treatments for insomnia in the elderly | 53 | 6,832 | None reported | None reported |
| (Lewis et al., 2012) | Meta-analysis | Self-help interventions for anxiety disorders | 31 | 615 + 545 | Not available in English | None reported |
| (Sheffield & Woods-Giscombé, 2016) | Systematic review | Perinatal yoga on women’s mental health and well-being | 13 | 667 | Not available in English | None reported |
| (D'Alò et al., 2020) | Rapid review | Using polyunsaturated fatty acids in children and adolescents with autism spectrum disorder | 14 | Not reported | No restrictions on language | None reported |
| (Khanbhai et al., 2019) | Mixed methods | Real-time patient experience feedback systems | 13 | Not reported | Not reported | Not primary or secondary care |
| (Fish et al., 2009) | Narrative review | Stepping exercise for cardiovascular fitness in women | Unclear | Not reported | Not reported | None reported |
| (Li et al., 2021) | Systematic review | Mindfulness-based interventions for people with recent onset psychosis | 8 | 118 | Not available in English | Not clinic or community |
| (Heynsbergh et al., 2018) | Systematic review | Technology-based interventions for informal cancer carers | 6 | Not reported | Not available in English | Not web-based or smartphone |
| (Apolinário-Hagen, 2019) | Narrative review | Internet-delivered psychological treatment options for panic disorder | 8 | 1,013 | Not available in English | None reported |
| (Mahoney & Veitch, 2018) | Integrative review | Interventions for maintaining nasogastric feeding after stroke | 7 | Not reported | Not available in English | None reported |
| (Maddocks et al., 2009) | Systematic review | Exercise for people with or cured of cancer | 65 | Not reported | Not reported | None reported |
| (Kayrouz et al., 2018) | Meta-analysis | Cognitive behavioural therapy for Arab adult populations experiencing anxiety, depression or post-traumatic stress disorder | 9 | 536 | Not available in Arabic, French or English  Not Arab | None reported |
| (Evans et al., 2020) | Mixed methods | Non-pharmacological interventions to reduce the symptoms of mild to moderate anxiety in pregnant women | 14 | 800 | Not available in English | None reported |
| (Sprogis et al., 2019) | Mixed methods | Wearable vital sign monitoring technologies in the acute care setting | 7 | 682 | Not available in English | Not hospital |
| (Hadgraft et al., 2018) | Mixed methods | Occupational sitting | 32 | 804 | Not available in English | Not workplace |
| (Apolinário-Hagen et al., 2017) | Scoping review | E-mental health treatment services for psychological problems | 4 | 3,336 | Not available in English | None reported |
| (Diepeveen et al., 2013) | Mixed methods | Government intervention to change health-related behaviours | 200 | Not reported | Not available in English | None reported |
| (Liew & Lee, 2019) | Network meta-analysis | Multicomponent interventions for caregiver depression in dementia | 33 | 6,138 | Not available in English or German | None reported |
| (Brooke-Sumner et al., 2015) | Systematic review | Psychosocial interventions for schizophrenia in low- and middle-income countries | 17 | Not reported | Not available in English  Not in LMIC | None reported |
| (Moulton-Perkins et al., 2020) | Mixed methods | Mindfulness-based cognitive therapy and mindfulness-based stress reduction via group videoconferencing | 10 | 789 | Not available in English | No exclusions |
| (Shek et al., 2021) | Mixed methods | Technology-based interventions for mental health support after stroke | 13 | Not reported | Not available in English | None reported |
| (Baker et al., 2020) | Rapid review | Web-based developmental surveillance programs | 20 | Not reported | Not available in English | Not internet |
| (Padmanathan & De Silva, 2013) | Mixed methods | Task-sharing for mental healthcare in low- and middle-income countries | 21 | Not reported | Not available in English  Not in LMIC | None reported |
| (Griffiths, 2020) | Mixed methods | Text messaging to support the delivery of physical health care in those suffering from a psychotic disorder | 11 | Not reported | Not available in English | Not mobile phone |
| (Zimmermann et al., 2018) | Mixed methods | Mindfulness based interventions in improving psychological well-being for adults with advanced cancer | 8 | 456 | Not available in English | None reported |
| (Gutiérrez-Cardona et al., 2017) | Mixed methods | Nasal douching in children with allergic rhinitis | 40 | Not reported | Not available in English | None reported |
| (Patel et al., 2020) | Qualitative meta synthesis | Digital health interventions for adults with depression | 24 | Not reported | Not available in English | Not community or primary or secondary care |
| (Toh et al., 2016) | Narrative review | Cognitive stimulation therapy for older adults with dementia | 12 | Not reported | Not available in English | No exclusions |
| (Bee et al., 2014) | Mixed methods | Community based interventions aimed at improving or maintaining quality of life in children of parents with serious mental illness | 10 | Not reported | Not available in English | Not non-residential |
| (Devereux-Fitzgerald et al., 2016) | Qualitative review | Physical activity interventions to older adults | 14 | Not reported | Not available in English | None reported |
| (Braz et al., 2017) | Systematic review | Vaginal smear self-collection for screening for cervical cancer | 19 | 18,202 | Not available in English, French, Italian, Portuguese or Spanish | None reported |
| (Kaltenthaler et al., 2008) | Systematic review | Computerized cognitive behaviour therapy for depression | 16 | Not reported | Not reported | None reported |
| (Hallam et al., 2021) | Meta analysis | Cognitive analytic therapy | 25 | Not reported | Not available in English | No exclusions |
| (Galdas et al., 2014) | Qualitative review | Self-management support interventions for men with long term conditions | 38 | Not reported | Not available in English | None reported |
| (Islam et al., 2012) | Narrative review | Primary healthcare outlets that target injecting drug users | 35 | Not reported | Not available in English | Not primary care |
| (Robinson et al., 2006) | Systematic review | Non-pharmacological interventions to prevent wandering in dementia | 10 | Not reported | No restrictions on language | Not a care environment |
| (O'Connor et al., 2018) | Meta analysis | Third-wave behavioral and cognitive eHealth treatments | 21 | 3,176 | Not available in English | None reported |
| (Stephen et al., 2018) | Integrative review | Nurse-led chronic disease management interventions in primary care | 11 | Not reported | Not available in English | Not primary care |
| (Tough et al., 2018) | Mixed methods | Exergaming among individuals with cancer | 8 | Not reported | Not available in English | None reported |
| (Faisal et al., 2020) | Scoping review | Smart oral multidose dispensing systems for medication adherence | 13 | 922 | Not available in English | None reported |
| (Wang et al., 2021) | Meta analysis | Curcumin for the treatment of depression or depressive symptoms | 10 | 594 | Not available in English | None reported |
| (Diffin et al., 2019) | Realist review | Personal health record for children and young people living with a complex health condition | 9 | Not reported | Not available in English | None reported |
| (Corbett et al., 2018) | Rapid review | Web-based interventions aiming to improve quality of life in cancer survivors | 16 | Not reported | None reported | None reported |
| (Nawaz et al., 2016) | Scoping review | Balance exergames in older adults | 19 | Not reported | Not available in English | None reported |
| (Holthe et al., 2018) | Mixed methods | Technology for community-dwelling older adults with mild cognitive impairment and dementia | 29 | 665 + 83 | Not available in English | None reported |
| (Rismawan et al., 2021) | Systematic review | Mobile application interventions for prevention or treatment of depression | 7 | Not reported | Not available in English or Bahasa Indonesia | None reported |
| (Chan & Honey, 2021) | Integrative review | Mobile digital apps for mental health | 17 | Not reported | Not available in English | None reported |
| (Griffin et al., 2019) | Systematic review | Vaginal ring among women in low-and-middle-income countries | 68 | Not reported | Not available in English  Not in LMIC | None reported |
| (Rus-Calafell et al., 2018) | Systematic review | Virtual reality in the assessment and treatment of psychosis | 50 | Not reported | Not available in English | None reported |
| (Gilbert et al., 2018) | Qualitative review | Videoconferencing in an orthopaedics setting | 4 | Not reported | Not available in English | Not orthopaedic setting |
| **FIDELITY** | | | | | | |
| **Citation details** | **Type of review** | **Healthcare intervention** | **Number of studies included** | **Total number of participants** | **Language or country exclusions** | **Setting exclusions** |
| (Dusenbury et al., 2003) | Literature review | Drug abuse prevention in school settings | Not reported | Not reported | Not reported | None reported |
| (Prowse et al., 2015) | Systematic review | Psychosocial clinical trials | 16 | Not reported | Not published in English | None reported |
| (Paulauskaite et al., 2019) | Systematic review | Complex interventions for people with intellectual disabilities and behaviours that challenge | 5 | 178 | Not published in English | None reported |
| (Lieberman-Betz, 2014) | Systematic review | Parent-mediated early communication intervention | 35 | 1,148 | None reported | None reported |
| (Gould et al., 2016) | Systematic review | School-based mindfulness and yoga interventions | 48 | Not reported | Not published in English | Not in school setting |
| (Lambert et al., 2017) | Systematic review | Individual level behaviour change interventions promoting physical activity among adults | 28 | Not reported | Not published in English | None reported |
| (Musuuza et al., 2016) | Systematic review | Interventions to improve hand hygiene of healthcare workers | 100 | Not reported | Not published in English | Not in healthcare setting |
| (Schinckus et al., 2014) | Systematic review | Diabetes self-management education programs | 20 | Not reported | Not published in English | None reported |
| (French et al., 2015) | Systematic review | Diagnosis and treatment of chronic cough in the adult | 23 | 3636 | Not published in English | Not outpatient or specialist or primary care |
| (Pérez et al., 2018) | Narrative review | Public health interventions in low-and middle-income countries | 90 | Not reported | Not published in English, Spanish or French  Not LMIC | None reported |
| (Brady et al., 2017) | Meta analysis | Chronic disease self-management education programs | 34 | 10,792 | Not published in English | Not community |
| (Garbacz et al., 2014) | Systematic review | Parent training programs for externalizing disorders in children and adolescents | 65 | Not reported | Not published in English | None reported |
| (Lamb et al., 2018) | Meta analysis | Use of assistive communications for children with autism spectrum disorder | 19 | 916 | Not published in English  Not in the USA | Not in school |
| (Maynard et al., 2013) | Systematic review | After-school program intervention for at-risk primary or secondary students | 55 | Not reported | Not published in English  Not in USA, Canada, UK, Ireland or Australia | None reported |
| (Lynas & Hawkins, 2017) | Systematic review | School-based child sexual abuse prevention programs | 17 | Not reported | Not published in English | Not primary or secondary school |
| (Parham et al., 2007) | Systematic review | Sensory integration | 34 | Not reported | Not published in English | None reported |
| (Johnson-Kozlow et al., 2008) | Systematic review | Secondhand smoking interventions | 29 | Not reported | Not published in English | None reported |
| (Gorman et al., 2021) | Systematic review | Interventions to reduce or prevent stress and/or anxiety from pregnancy up to 2 years postpartum | 16 | Not reported | None reported | None reported |
| (Neely et al., 2017) | Systematic review | Autism focused interventionists coached via telepractice | 19 | 155 | None reported | Not delivered by ICT |
| (Keller et al., 2009) | Systematic review | Theory in physical activity | 15 | Not reported | Not published in English | None reported |
| (Toomey et al., 2015) | Rapid review | Physiotherapist-delivered group education and exercise interventions to promote self-management in people with osteoarthritis and chronic low back pain | 22 | Not reported | Not published in English | None reported |
| (Brady et al., 2019) | Systematic review | Behavioural interventions for people with intellectual and developmental disabilities | 20 | 100 | Not published in English | No exclusions |
| (JaKa et al., 2016) | Meta analysis | Behavioural paediatric obesity interventions | 193 | Not reported | Not published in English | None reported |
| (Preyde & Burnham, 2011) | Systematic review | Psychosocial oncology | 28 | Not reported | Not published in English | None reported |
| (Chang et al., 2019) | Method review | Qigong | 32 | Not reported | Not published in English | None reported |
| (Toomey et al., 2019) | Systematic review | Infant feeding behavioral interventions to prevent childhood obesity | 10 | Not reported | No language exclusions | Not a healthcare setting or by a health professional |
| (MacDonald et al., 2016) | Systematic review | Condom use among middle-aged and older adults | 5 | 565 | Not published in English | None reported |
| (Rojas-Andrade & Bahamondes, 2019) | Systematic review | School-based mental health programs | 31 | Not reported | None reported | Not in a school |
| (Schaap et al., 2018) | Systematic review | School-based obesity prevention programmes | 63 | Not reported | Not published in English | Not in a school |
| (Bennett et al., 2011) | Method review | Transcutaneous electric nerve stimulation for pain | 38 | 2,268 | None reported | Not acute or chronic setting |
| (Bartlett Ellis et al., 2017) | Systematic review | Pillbox in medication adherence | 38 | Not reported | Not published in English | None reported |
| (Murta et al., 2007) | Systematic review | Occupational stress management | 52 | Not reported | Not published in English | Not a workplace |
| (Ang et al., 2018) | Systematic review | Palliative care complex interventions | 20 | 8,426 | Not published in English | None reported |
| (Salamh et al., 2019) | Meta analysis | Interventions used to reduce posterior shoulder tightness | 13 | Not reported | Not published in English | None reported |
| (Yeary et al., 2012) | Systematic review | Church-based health interventions | 67 | Not reported | None reported | Not a church setting |
| (Schober et al., 2013) | Systematic review | Primary prevention programmes for eating disorders in schools | 38 | Not reported | None reported | Not in a classroom setting |
| (Salamh et al., 2017) | Meta analysis | Manual therapy to the knee | 12 | Not reported | Not published in English | None reported |
| (Brogan et al., 2019) | Systematic review | Treatment fidelity in aphasia randomised controlled trials | 42 | Not reported | Not published in English | None reported |
| (McArthur et al., 2012) | Systematic review | Psychosocial intervention for children and adolescents with comorbid problems | 10 | Not reported | Not published in English | None reported |
| (Naleppa & Cagle, 2010) | Systematic review | Social work interventions | 63 | Not reported | None reported | None reported |
| (Hinckley & Douglas, 2013) |  | Aphasia treatment studies | 149 | Not reported | None reported | None reported |
| **FEASIBILITY** | | | | | | |
| **Citation details** | **Type of review** | **Healthcare intervention** | **Number of studies included** | **Total number of participants** | **Language or country exclusions** | **Setting exclusions** |
| (Singh et al., 2018) | Meta analysis | Exercise in women with stage II+ breast cancer | 61 | Not reported | Not published in Englished | None reported |
| (Bakker et al., 2019) | Systematic review | Mobile technologies in clinical research | 275 | Not reported | None reported | None reported |
| (Brebner et al., 2006) | Systematic review | Accident and emergency teleconsultation | 31 | Not reported | None reported | Not primary care |
| (Magalhães et al., 2014) | Meta analysis | Cognitive interventions for multiple sclerosis | 5 | 39 | No language restrictions | None reported |
| (Njoroge et al., 2017) | Systematic review | eHealth and mHealth | 69 | Not reported | Not in Kenya | Not ICT |
| (Cordner et al., 2021) | Systematic review | Ballistic resistance training in adults with neurologic conditions | 13 | 279 | Not published in Englished | None reported |
| (Cole, 2008) | Systematic review | Brief interventions to prevent depression in older subjects | 14 | Not reported | Not published in English or French | None reported |
| (Cole & Dendukuri, 2004) | Systematic review | Brief interventions to prevent depression in older subjects | 10 | Not reported | Not published in English or French | None reported |
| (Schröder, van Criekinge, et al., 2019) | Systematic review | Tele-rehabilitation and virtual reality-based balance training | 7 | 120 | Not published in English, Dutch, German or French | Not in home |
| (Seitz et al., 2012) | Systematic review | Nonpharmacological interventions for neuropsychiatric symptoms of dementia in long term care | 40 | 3,519 | Not published in Englished | Not in long term care |
| (Droste et al., 2014) | Systematic review | Emergency department data sharing to reduce alcohol-related violence | 8 | Not reported | Not published in Englished | Not in emergency department |
| (Agarwal et al., 2015) | Systematic review | mHealth strategies by frontline health workers in developing countries | 42 | Not reported | Not published in Englished  Not in a developing country | Not mobile device |
| (Singh, Hayes, et al., 2020) | Systematic review | Exercise and colorectal cancer | 19 | Not reported | Not published in Englished | Not reported |
| (Singh, Spence, et al., 2020) | Systematic review | Exercise for individuals with lung cancer | 32 | 2109 | Not published in Englished | None reported |
| (Kosse et al., 2013) | Systematic review | Early physical rehabilitation programs for geriatric hospitalized patients | 15 | Not reported | Not published in Englished | None reported |
| (Chipps et al., 2012) | Systematic review of systematic reviews | Telepsychiatry in resource constrained environments | 10 | Not reported | Not published in English | Not telepsychiatry |
| (Miller et al., 2014) | Systematic review | Virtual reality and gaming system use at home by older adults for enabling physical activity | 14 | Not reported | Not published in Englished | Not at home |
| (Scholz et al., 2020) | Systematic review | Psychological interventions to reduce inattention symptoms in adults with ADHD | 19 | Not reported | None reported | None reported |
| (Beckers et al., 2020) | Systematic review | Home-based therapy programmes for children with cerebral palsy | 92 | Not reported | No language restrictions | Not at home |
| (Weber et al., 2018) | Systematic review | Integrating functional exercise into daily life of older adults | 14 | Not reported | No language restrictions | None reported |
| (Schröder, Truijen, et al., 2019) | Meta analysis | Repetitive gait training early after stroke | 15 | 915 | Not published in English, German or Dutch | Non reported |
| (Cole et al., 2000) | Systematic review | Treatments for depression in elderly medical inpatients | 15 | Not reported | Not published in English or French | Not acute care |
| (Ridgers et al., 2016) | Systematic review | Using wearable activity trackers in youth | 5 | Not reported | Not published in English | None reported |
| (Nesvåg & McKay, 2018) | Systematic review | Digital interventions to support people in recovery from substance use disorders | 43 | Not reported | None reported | None reported |
| (Klausen et al., 2016) | Systematic review | Eye tracking in critical care environments | 8 | Not reported | Not published in English | Not critical care |
| (Delgado-Floody et al., 2019) | Systematic review | High-intensity interval training in physical education programs to improve body composition and cardiorespiratory capacity of overweight and obese children | 6 | Not reported | Not published in English | None reported |
| (Jonker et al., 2020) | Systematic review | eHealth interventions for older surgical patients | 7 | 223 | No language restrictions | Not mobile technology |
| (Gough et al., 2020) | Systematic review | Remotely supervised transcranial direct current stimulation and cognitive remediation | 39 | Not reported | Not published in English | Not at home |
| (Soneson et al., 2020) | Systematic review | School-based identification of children and adolescents experiencing, or at-risk of developing, mental health difficulties | 33 | Not reported | Not published in English | Not at school |
| (Moug et al., 2017) | Systematic review | Lifestyle interventions in patients with colorectal cancer | 14 | Not reported | No language restrictions | None reported |
| (Emerson et al., 2019) | Systematic review | Mindfulness intervention in schools | 31 | Not reported | Not published in English | Not in school |
| (Horrell et al., 2020) | Qualitative review | Physical activity in the reduction of and abstinence from alcohol and other drug use | 20 | Not reported | Not published in English | No exclusions |
| (Learmonth & Motl, 2018) | Scoping review | Physical activity involving persons with multiple sclerosis | 25 | Not reported | Not published in English | None reported |
| (Alves et al., 2013) | Systematic review | Cognitive interventions in Alzheimer disease | 4 | Not reported | No language restrictions | None reported |
| (Peltea et al., 2016) | Systematic review | Knee ultrasound assessment and interventions | 9 | 1,052 | Not published in English | None reported |
| (Tarabay et al., 2016) | Systematic review | Injection safety devices in healthcare settings | 14 | Not reported | No language restrictions | Not healthcare settings |
| (Mayer et al., 2020) | Systematic review | Physical rehabilitation and active mobilization in patients requiring continuous renal replacement therapy | 15 | 437 | Not published in English | Not in ICU |
| (Heywood et al., 2017) | Systematic review | Exercise interventions in patients with advanced cancer | 25 | 1,088 | Not published in English | None reported |
| (Terrens et al., 2018) | Systematic review | Aquatic physiotherapy for people with Parkinson’s disease | 10 | 119 | Not published in English | Not in a heated pool |
| (Kininger et al., 2018) | Systematic review | School-based modular therapy | 7 | Not reported | Not published in English | Not in school |
| (Grimshaw et al., 2016) | Systematic review | Physical activity interventions during the intense treatment phase for children and adolescents with cancer | 12 | Not reported | Not published in English | None reported |
| (Scott et al., 2011) | Systematic review | Emergency department-based hypertension screening | 4 | Not reported | None reported | Not in emergency department setting |
| (Zaga et al., 2019) | Systematic review | Communication interventions with mechanically ventilated intensive care unit patients | 48 | Not reported | Not published in English | Not in intensive care |
| (Booth et al., 2015) | Systematic review | Group clinics for patients with chronic conditions | 44 | Not reported | Not published in English | Not in group setting |
| (Ruzicka et al., 2014) | Systematic review | Sodium reduction strategies to treat hypertension in primary care settings | 6 | Not reported | No language restrictions | Not in primary care |
| (Troy et al., 2018) | Systematic review | Parenting and family support programs delivered in the criminal justice system | 29 | Not reported | Not published in English | Not in the criminal justice system |
| **SUSTAINABILITY** | | | | | | |
| **Citation details** | **Type of review** | **Healthcare intervention** | **Number of studies included** | **Total number of participants** | **Language or country exclusions** | **Setting exclusions** |
| (Cassar et al., 2019) | Systematic review | School-based activity and sedentary behaviour | 27 | Not reported | Not published in English | Not in a school setting |
| (O'Shea et al., 2020) | Systematic review | Abortion services | 23 | 1,016 | Not published in English  Not in OECD countries | None reported |
| (Hearld et al., 2016) | Systematic review | Community health collaboratives | 42 | Not reported | Not reported | None reported |
| (Wright et al., 2004) | Systematic review | Home treatment services | 91 | Not reported | None reported | In home setting |
| (Flämig et al., 2019) | Scoping review | ART adherence clubs in the Western Cape of South Africa | 6 | Not reported | Not published in English  Not South Africa | None reported |
| (Kiberu et al., 2017) | Literature review | Sustainable e-Health programmes in Uganda | 48 | Not reported | Not published in English  Not Uganda | Not e-Health |
| (Braithwaite et al., 2020) | Systematic integrative review | Healthcare system improvements, programmes and interventions | 92 | Not reported | Not published in English | Not healthcare settings |
| (Shrime et al., 2015) | Systematic review | Charitable platforms in global surgery | 104 | None reported | No language restrictions  Not LMIC | None reported |
| (Pallas et al., 2013) | Systematic review | Community health workers in Low-and middle-income countries | 19 | Not reported | Not published in English, Spanish, French or Portuguese  Not LMIC | Not community |
| (Ishola & Cekan, 2019) | Literature review | Health programmes | 4 | Not reported | Not published in English  Not in Nigeria | Not reported |
| (Hailemariam et al., 2019) | Systematic review | Evidence-based interventions | 26 | Not reported | Not published in English | Not community based |
| (Francis et al., 2016) | Scoping review | Chronic disease health programmes | 42 | Not reported | Not published in English | Not primary care or hospitals or mental health centres or community health |
| (Woodnutt, 2018) | Systematic literature review | Lean in NHS hospitals | 12 | Not reported | Not published in English  Not in UK | None reported |
| (Mok et al., 2019) | Systematic review | Childhood obesity interventions | 8 | Not reported | Not published in English | None reported |
| (Lovarini et al., 2013) | Systematic review | Community-based fall prevention programs | 19 | Not reported | Not published in English | Not community |
| (James et al., 2021) | Systematic review | Video consulting in health care | 13 | Not reported | Not published in English | Not synchronous video-based conferencing |
| (Ament et al., 2015) | Systematic review | Professional adherence to clinical practice guidelines in medical care | 14 | Not reported | None reported | None reported |
| (Crespo-Gonzalez et al., 2020) | Systematic review | Innovations in professional pharmacy services | 132 | Not reported | Not published with Latin alphabet | None reported |
| (Lauckner & Whitten, 2016) | Systematic review | Telepsychiatry Program | 68 | Not reported | None reported | Not delivered via tele-mode |
| (de Vries & Pool, 2017) | Systematic review | Community health resources and community and lay health worker programs in lower-income countries | 32 | Not reported | Not published in English  Not LIC | Not community |
| (Cowie et al., 2020) | Systematic review | Hospital-based interventions | 32 | Not reported | Not published in English | Not a hospital setting |
| (Bradford et al., 2016) | Systematic review | Telehealth services in rural and remote Australia | 116 | Not reported | Not published in English  Not rural or remote Australia | Not telehealth |
| (Iwelunmor et al., 2016) | Systematic review | Health interventions implemented in sub-Saharan Africa | 41 | Not reported | Not published in English  Not sub-Saharan | None reported |
| (Herlitz et al., 2020) | Systematic review | Public health interventions in schools | 24 | Not reported | Not published in English | Not in a school setting |
| (Flynn et al., 2018) | Realist review | Lean in pediatric healthcare | 11 | Not reported | Not published in English | Not in a healthcare setting |
| **SCALABILITY** | | | | | | |
| **Citation details** | **Type of review** | **Healthcare intervention** | **Number of studies included** | **Total number of participants** | **Language or country exclusions** | **Setting exclusions** |
| (Chapman et al., 2010) | Systematic review | Breastfeeding peer counselling | 7 | Not reported | Not published in English, Spanish, French or Portuguese | None reported |
| (Troup et al., 2021) | Systematic review | Mental health and psychosocial support interventions in low- and middle-income countries for populations affected by humanitarian crises | 14 | Not reported | Not published in English  Not in LMIC | Not a post conflict setting |
| (Carroll et al., 2020) | Systematic review | Breastfeeding interventions | 45 | Not reported | Not published in English, Spanish or Portuguese | None reported |
| (Vassall & Compernolle, 2006) | Systematic review | HIV/AIDS and tuberculosis interventions in sub-Saharan Africa | 19 | Not reported | Not published in English  Not sub-Saharan Africa | None reported |
| (Ben Charif et al., 2017) | Systematic review | Evidence-based practices in primary care | 14 | Not reported | No language restrictions  No country restriction | Not in primary care |
| (McCrabb et al., 2019) | Systematic review | Evidence-based obesity interventions | 10 | Not reported | None reported | Not community or nonclinical settings |
| (Lane et al., 2021) | Systematic review | Physical activity interventions | 10 | Not reported | None reported | Medical/clinical setting |
| (Wiessing et al., 2014) | Systematic review | Hepatitis C virus infection among people who inject drugs in Europe | 144 | 6,488 | No language restrictions  Not Europe | None reported |
| (Bulthuis et al., 2020) | Qualitative review | Public health interventions in low- and middle-income countries | 27 | Not reported | Not in English  Not LMIC | None reported |

**References**

1. Agarwal S, Perry HB, Long LA, Labrique AB. Evidence on feasibility and effective use of mHealth strategies by frontline health workers in developing countries: systematic review. Tropical medicine & international health : TM & IH. 2015;20(8):1003-14.
2. Alves J, Magalhaes R, Thomas RE, Goncalves OF, Petrosyan A, Sampaio A. Is there evidence for cognitive intervention in Alzheimer disease? A systematic review of efficacy, feasibility, and cost-effectiveness. Alzheimer disease and associated disorders. 2013;27(3):195-203.
3. Ament SM, de Groot JJ, Maessen JM, Dirksen CD, van der Weijden T, Kleijnen J. Sustainability of professionals' adherence to clinical practice guidelines in medical care: a systematic review. BMJ open. 2015;5(12):e008073.
4. Andrews G, Basu A, Cuijpers P, Craske MG, McEvoy P, English CL, et al. Computer therapy for the anxiety and depression disorders is effective, acceptable and practical health care: An updated meta-analysis. J Anxiety Disord. 2018;55:70-8.
5. Ang K, Hepgul N, Gao W, Higginson IJ. Strategies used in improving and assessing the level of reporting of implementation fidelity in randomised controlled trials of palliative care complex interventions: A systematic review. Palliat Med. 2018;32(2):500-16.
6. Aparicio LVM, Guarienti F, Razza LB, Carvalho AF, Fregni F, Brunoni AR. A Systematic Review on the Acceptability and Tolerability of Transcranial Direct Current Stimulation Treatment in Neuropsychiatry Trials. Brain Stimul. 2016;9(5):671-81.
7. Apolinario-Hagen J. Internet-Delivered Psychological Treatment Options for Panic Disorder: A Review on Their Efficacy and Acceptability. Psychiatry Investig. 2019;16(1):37-49.
8. Apolinario-Hagen J, Kemper J, Sturmer C. Public Acceptability of E-Mental Health Treatment Services for Psychological Problems: A Scoping Review. JMIR Ment Health. 2017;4(2):e10.
9. Babiano-Espinosa L, Wolters LH, Weidle B, Op de Beek V, Pedersen SA, Compton S, et al. Acceptability, feasibility, and efficacy of Internet cognitive behavioral therapy (iCBT) for pediatric obsessive-compulsive disorder: a systematic review. Syst Rev. 2019;8(1):284.
10. Baker J, Kohlhoff J, Onobrakpor SI, Woolfenden S, Smith R, Knebel C, et al. The Acceptability and Effectiveness of Web-Based Developmental Surveillance Programs: Rapid Review. JMIR Mhealth Uhealth. 2020;8(4):e16085.
11. Bakker JP, Goldsack JC, Clarke M, Coravos A, Geoghegan C, Godfrey A, et al. A systematic review of feasibility studies promoting the use of mobile technologies in clinical research. NPJ digital medicine. 2019;2:47.
12. Bartlett Ellis RJ, Knisely MR, Boyer K, Pike C. Pillbox intervention fidelity in medication adherence research: A systematic review. Nurs Outlook. 2017;65(4):464-76.
13. Bautista T, James D, Amaro H. Acceptability of mindfulness-based interventions for substance use disorder: A systematic review. Complement Ther Clin Pract. 2019;35:201-7.
14. Beckers L, Geijen MME, Kleijnen J, E AAR, M LAPS, R JEMS, et al. Feasibility and effectiveness of home-based therapy programmes for children with cerebral palsy: a systematic review. BMJ open. 2020;10(10):e035454.
15. Bee P, Bower P, Byford S, Churchill R, Calam R, Stallard P, et al. The clinical effectiveness, cost-effectiveness and acceptability of community-based interventions aimed at improving or maintaining quality of life in children of parents with serious mental illness: a systematic review. Health Technol Assess. 2014;18(8):1-250.
16. Ben Charif A, Zomahoun HTV, LeBlanc A, Langlois L, Wolfenden L, Yoong SL, et al. Effective strategies for scaling up evidence-based practices in primary care: a systematic review. Implementation science : IS. 2017;12(1):139.
17. Bennett MI, Hughes N, Johnson MI. Methodological quality in randomised controlled trials of transcutaneous electric nerve stimulation for pain: low fidelity may explain negative findings. Pain. 2011;152(6):1226-32.
18. Berlim MT, McGirr A, Van den Eynde F, Fleck MP, Giacobbe P. Effectiveness and acceptability of deep brain stimulation (DBS) of the subgenual cingulate cortex for treatment-resistant depression: a systematic review and exploratory meta-analysis. J Affect Disord. 2014;159:31-8.
19. Berlim MT, Van den Eynde F, Daskalakis ZJ. A systematic review and meta-analysis on the efficacy and acceptability of bilateral repetitive transcranial magnetic stimulation (rTMS) for treating major depression. Psychol Med. 2013;43(11):2245-54.
20. Berlim MT, Van den Eynde F, Jeff Daskalakis Z. Clinically meaningful efficacy and acceptability of low-frequency repetitive transcranial magnetic stimulation (rTMS) for treating primary major depression: a meta-analysis of randomized, double-blind and sham-controlled trials. Neuropsychopharmacology. 2013;38(4):543-51.
21. Bernardy K, Klose P, Welsch P, Hauser W. Efficacy, acceptability and safety of cognitive behavioural therapies in fibromyalgia syndrome - A systematic review and meta-analysis of randomized controlled trials. Eur J Pain. 2018;22(2):242-60.
22. Berry N, Lobban F, Emsley R, Bucci S. Acceptability of Interventions Delivered Online and Through Mobile Phones for People Who Experience Severe Mental Health Problems: A Systematic Review. J Med Internet Res. 2016;18(5):e121.
23. Booth A, Cantrell A, Preston L, Chambers D, Goyder E. Health Services and Delivery Research. What is the evidence for the effectiveness, appropriateness and feasibility of group clinics for patients with chronic conditions? A systematic review. Health Services and Delivery Research. Southampton (UK): NIHR Journals Library
24. Bradford NK, Caffery LJ, Smith AC. Telehealth services in rural and remote Australia: a systematic review of models of care and factors influencing success and sustainability. Rural Remote Health. 2016;16(4):3808.
25. Brady L, Padden C, McGill P. Improving procedural fidelity of behavioural interventions for people with intellectual and developmental disabilities: A systematic review. J Appl Res Intellect Disabil. 2019;32(4):762-78.
26. Brady TJ, Murphy LB, O'Colmain BJ, Hobson RD. Do Program Implementation Factors or Fidelity Affect Chronic Disease Self-Management Education Programs' Outcomes? Am J Health Promot. 2017;31(5):422-5.
27. Braithwaite J, Ludlow K, Testa L, Herkes J, Augustsson H, Lamprell G, et al. Built to last? The sustainability of healthcare system improvements, programmes and interventions: a systematic integrative review. BMJ open. 2020;10(6):e036453.
28. Braz NS, Lorenzi NP, Sorpreso IC, Aguiar LM, Baracat EC, Soares-Junior JM. The acceptability of vaginal smear self-collection for screening for cervical cancer: a systematic review. Clinics (Sao Paulo). 2017;72(3):183-7.
29. Brebner JA, Brebner EM, Ruddick-Bracken H. Accident and emergency teleconsultation for primary care--a systematic review of technical feasibility, clinical effectiveness, cost effectiveness and level of local management. Journal of telemedicine and telecare. 2006;12 Suppl 1:5-8.
30. Brogan E, Ciccone N, Godecke E. Treatment fidelity in aphasia randomised controlled trials. Aphasiology. 2019;33(7):759-79.
31. Brooke-Sumner C, Petersen I, Asher L, Mall S, Egbe CO, Lund C. Systematic review of feasibility and acceptability of psychosocial interventions for schizophrenia in low and middle income countries. BMC Psychiatry. 2015;15:19.
32. Buckingham P, Moulton JE, Subasinghe AK, Amos N, Mazza D. Acceptability of immediate postpartum and post-abortion long-acting reversible contraception provision to adolescents: A systematic review. Acta Obstet Gynecol Scand. 2021;100(4):629-40.
33. Bulthuis SE, Kok MC, Raven J, Dieleman MA. Factors influencing the scale-up of public health interventions in low- and middle-income countries: a qualitative systematic literature review. Health policy and planning. 2020;35(2):219-34.
34. Carroll G, Safon C, Buccini G, Vilar-Compte M, Teruel G, Pérez-Escamilla R. A systematic review of costing studies for implementing and scaling-up breastfeeding interventions: what do we know and what are the gaps? Health policy and planning. 2020;35(4):461-501.
35. Cassar S, Salmon J, Timperio A, Naylor PJ, van Nassau F, Contardo Ayala AM, et al. Adoption, implementation and sustainability of school-based physical activity and sedentary behaviour interventions in real-world settings: a systematic review. Int J Behav Nutr Phys Act. 2019;16(1):120.
36. Chan AHY, Honey MLL. User perceptions of mobile digital apps for mental health: Acceptability and usability - An integrative review. J Psychiatr Ment Health Nurs. 2021.
37. Chang PS, Chao AM, Jang M, Lu YYF. Intervention fidelity in Qigong randomized controlled trials: A method review. Geriatr Nurs. 2019;40(1):84-90.
38. Chapman DJ, Morel K, Anderson AK, Damio G, Pérez-Escamilla R. Breastfeeding peer counseling: from efficacy through scale-up. J Hum Lact. 2010;26(3):314-26.
39. Chipps J, Brysiewicz P, Mars M. Effectiveness and feasibility of telepsychiatry in resource constrained environments? A systematic review of the evidence. African journal of psychiatry. 2012;15(4):235-43.
40. Chou PH, Tseng PT, Wu YC, Chang JP, Tu YK, Stubbs B, et al. Efficacy and acceptability of different interventions for acrophobia: A network meta-analysis of randomised controlled trials. J Affect Disord. 2021;282:786-94.
41. Chu CS, Li CT, Brunoni AR, Yang FC, Tseng PT, Tu YK, et al. Cognitive effects and acceptability of non-invasive brain stimulation on Alzheimer's disease and mild cognitive impairment: a component network meta-analysis. J Neurol Neurosurg Psychiatry. 2021;92(2):195-203.
42. Cleeve A, Fonhus MS, Lavelanet A. A systematic review of the effectiveness, safety, and acceptability of medical management of intrauterine fetal death at 14-28 weeks of gestation. Int J Gynaecol Obstet. 2019;147(3):301-12.
43. Cole MG. Brief interventions to prevent depression in older subjects: a systematic review of feasibility and effectiveness. The American journal of geriatric psychiatry : official journal of the American Association for Geriatric Psychiatry. 2008;16(6):435-43.
44. Cole MG, Dendukuri N. The feasibility and effectiveness of brief interventions to prevent depression in older subjects: a systematic review. Int J Geriatr Psychiatry. 2004;19(11):1019-25.
45. Cole MG, Elie LM, McCusker J, Bellavance F, Mansour A. Feasibility and effectiveness of treatments for depression in elderly medical inpatients: a systematic review. International psychogeriatrics. 2000;12(4):453-61.
46. Coorey GM, Neubeck L, Mulley J, Redfern J. Effectiveness, acceptability and usefulness of mobile applications for cardiovascular disease self-management: Systematic review with meta-synthesis of quantitative and qualitative data. Eur J Prev Cardiol. 2018;25(5):505-21.
47. Corbett T, Singh K, Payne L, Bradbury K, Foster C, Watson E, et al. Understanding acceptability of and engagement with Web-based interventions aiming to improve quality of life in cancer survivors: A synthesis of current research. Psychooncology. 2018;27(1):22-33.
48. Cordner T, Egerton T, Schubert K, Wijesinghe T, Williams G. Ballistic Resistance Training: Feasibility, Safety, and Effectiveness for Improving Mobility in Adults With Neurologic Conditions: A Systematic Review. Archives of physical medicine and rehabilitation. 2021;102(4):735-51.
49. Cowie J, Nicoll A, Dimova ED, Campbell P, Duncan EA. The barriers and facilitators influencing the sustainability of hospital-based interventions: a systematic review. BMC Health Serv Res. 2020;20(1):588.
50. Craig C, Hiskey S, Spector A. Compassion focused therapy: a systematic review of its effectiveness and acceptability in clinical populations. Expert Rev Neurother. 2020;20(4):385-400.
51. Cremers G, Taylor E, Hodge L, Quigley A. Effectiveness and Acceptability of Low-intensity Psychological Interventions on the Well-being of Older Adults: A Systematic Review. Clin Gerontol. 2019:1-21.
52. Crespo-Gonzalez C, Benrimoj SI, Scerri M, Garcia-Cardenas V. Sustainability of innovations in healthcare: A systematic review and conceptual framework for professional pharmacy services. Res Social Adm Pharm. 2020;16(10):1331-43.
53. Cuijpers P, Noma H, Karyotaki E, Cipriani A, Furukawa TA. Effectiveness and Acceptability of Cognitive Behavior Therapy Delivery Formats in Adults With Depression: A Network Meta-analysis. JAMA Psychiatry. 2019;76(7):700-7.
54. D'Alo GL, De Crescenzo F, Minozzi S, Morgano GP, Mitrova Z, Scattoni ML, et al. Equity, acceptability and feasibility of using polyunsaturated fatty acids in children and adolescents with autism spectrum disorder: a rapid systematic review. Health Qual Life Outcomes. 2020;18(1):101.
55. Dai Y, Livesley J. A mixed-method systematic review of the effectiveness and acceptability of preoperative psychological preparation programmes to reduce paediatric preoperative anxiety in elective surgery. J Adv Nurs. 2018.
56. Davies C, Radua J, Cipriani A, Stahl D, Provenzani U, McGuire P, et al. Efficacy and Acceptability of Interventions for Attenuated Positive Psychotic Symptoms in Individuals at Clinical High Risk of Psychosis: A Network Meta-Analysis. Front Psychiatry. 2018;9:187.
57. Davis MM, Freeman M, Kaye J, Vuckovic N, Buckley DI. A systematic review of clinician and staff views on the acceptability of incorporating remote monitoring technology into primary care. Telemed J E Health. 2014;20(5):428-38.
58. de Barros SF, Cardoso MA. Adherence to and acceptability of home fortification with vitamins and minerals in children aged 6 to 23 months: a systematic review. BMC Public Health. 2016;16:299.
59. De Crescenzo F, Ciabattini M, D'Alo GL, De Giorgi R, Del Giovane C, Cassar C, et al. Comparative efficacy and acceptability of psychosocial interventions for individuals with cocaine and amphetamine addiction: A systematic review and network meta-analysis. PLoS Med. 2018;15(12):e1002715.
60. de Vries DH, Pool R. The Influence of Community Health Resources on Effectiveness and Sustainability of Community and Lay Health Worker Programs in Lower-Income Countries: A Systematic Review. PLoS One. 2017;12(1):e0170217.
61. Delgado-Floody P, Latorre-Roman P, Jerez-Mayorga D, Caamano-Navarrete F, Garcia-Pinillos F. Feasibility of incorporating high-intensity interval training into physical education programs to improve body composition and cardiorespiratory capacity of overweight and obese children: A systematic review. Journal of exercise science and fitness. 2019;17(2):35-40.
62. Devereux-Fitzgerald A, Powell R, Dewhurst A, French DP. The acceptability of physical activity interventions to older adults: A systematic review and meta-synthesis. Soc Sci Med. 2016;158:14-23.
63. Diepeveen S, Ling T, Suhrcke M, Roland M, Marteau TM. Public acceptability of government intervention to change health-related behaviours: a systematic review and narrative synthesis. BMC Public Health. 2013;13:756.
64. Diffin J, Byrne B, Kerr H, Price J, Abbott A, McLaughlin D, et al. The usefulness and acceptability of a personal health record to children and young people living with a complex health condition: A realist review of the literature. Child Care Health Dev. 2019;45(3):313-32.
65. Driedger M, Mayhew A, Welch V, Agbata E, Gruner D, Greenaway C, et al. Accessibility and Acceptability of Infectious Disease Interventions Among Migrants in the EU/EEA: A CERQual Systematic Review. Int J Environ Res Public Health. 2018;15(11).
66. Droste N, Miller P, Baker T. Review article: Emergency department data sharing to reduce alcohol-related violence: a systematic review of the feasibility and effectiveness of community-level interventions. Emerg Med Australas. 2014;26(4):326-35.
67. Dusenbury L, Brannigan R, Falco M, Hansen WB. A review of research on fidelity of implementation: implications for drug abuse prevention in school settings. Health Educ Res. 2003;18(2):237-56.
68. Emerson LM, de Diaz NN, Sherwood A, Waters A, Farrell L. Mindfulness interventions in schools: Integrity and feasibility of implementation. International Journal of Behavioral Development. 2020;44(1):62-75.
69. Evans K, Spiby H, Morrell JC. Non-pharmacological interventions to reduce the symptoms of mild to moderate anxiety in pregnant women. A systematic review and narrative synthesis of women's views on the acceptability of and satisfaction with interventions. Arch Womens Ment Health. 2020;23(1):11-28.
70. Faisal S, Ivo J, Lee C, Carter C, Patel T. The Usability, Acceptability, and Functionality of Smart Oral Multidose Dispensing Systems for Medication Adherence: A Scoping Review. J Pharm Pract. 2020:897190020977756.
71. Faulkner SM, Dijk DJ, Drake RJ, Bee PE. Adherence and acceptability of light therapies to improve sleep in intrinsic circadian rhythm sleep disorders and neuropsychiatric illness: a systematic review. Sleep Health. 2020;6(5):690-701.
72. Figueroa C, Johnson C, Verster A, Baggaley R. Attitudes and Acceptability on HIV Self-testing Among Key Populations: A Literature Review. AIDS Behav. 2015;19(11):1949-65.
73. Fish AF, Christman SK, Frid DJ, Smith BA, Bryant CX. Feasibility and acceptability of stepping exercise for cardiovascular fitness in women. Appl Nurs Res. 2009;22(4):274-9.
74. Flämig K, Decroo T, van den Borne B, van de Pas R. ART adherence clubs in the Western Cape of South Africa: what does the sustainability framework tell us? A scoping literature review. J Int AIDS Soc. 2019;22(3):e25235.
75. Flynn R, Newton AS, Rotter T, Hartfield D, Walton S, Fiander M, et al. The sustainability of Lean in pediatric healthcare: a realist review. Syst Rev. 2018;7(1):137.
76. Forbes CC, Finlay A, McIntosh M, Siddiquee S, Short CE. A systematic review of the feasibility, acceptability, and efficacy of online supportive care interventions targeting men with a history of prostate cancer. J Cancer Surviv. 2019;13(1):75-96.
77. Francis L, Dunt D, Cadilhac DA. How is the sustainability of chronic disease health programmes empirically measured in hospital and related healthcare services?-a scoping review. BMJ open. 2016;6(5):e010944.
78. French CT, Diekemper RL, Irwin RS, Adams TM, Altman KW, Barker AF, et al. Assessment of Intervention Fidelity and Recommendations for Researchers Conducting Studies on the Diagnosis and Treatment of Chronic Cough in the Adult: CHEST Guideline and Expert Panel Report. Chest. 2015;148(1):32-54.
79. Galdas P, Darwin Z, Kidd L, Blickem C, McPherson K, Hunt K, et al. The accessibility and acceptability of self-management support interventions for men with long term conditions: a systematic review and meta-synthesis of qualitative studies. BMC Public Health. 2014;14:1230.
80. Gallo MF, Kilbourne-Brook M, Coffey PS. A review of the effectiveness and acceptability of the female condom for dual protection. Sex Health. 2012;9(1):18-26.
81. Garbacz LL, Brown DM, Spee GA, Polo AJ, Budd KS. Establishing treatment fidelity in evidence-based parent training programs for externalizing disorders in children and adolescents. Clin Child Fam Psychol Rev. 2014;17(3):230-47.
82. Gerger H, Werner CP, Gaab J, Cuijpers P. Comparative efficacy and acceptability of expressive writing treatments compared with psychotherapy, other writing treatments, and waiting list control for adult trauma survivors: a systematic review and network meta-analysis. Psychol Med. 2021:1-13.
83. Gilbert AW, Jaggi A, May CR. What is the patient acceptability of real time 1:1 videoconferencing in an orthopaedics setting? A systematic review. Physiotherapy. 2018;104(2):178-86.
84. Gilbey D, Morgan H, Lin A, Perry Y. Effectiveness, Acceptability, and Feasibility of Digital Health Interventions for LGBTIQ+ Young People: Systematic Review. J Med Internet Res. 2020;22(12):e20158.
85. Giles EL, Robalino S, Sniehotta FF, Adams J, McColl E. Acceptability of financial incentives for encouraging uptake of healthy behaviours: A critical review using systematic methods. Prev Med. 2015;73:145-58.
86. Goldberg SB, Riordan KM, Sun S, Kearney DJ, Simpson TL. Efficacy and acceptability of mindfulness-based interventions for military veterans: A systematic review and meta-analysis. J Psychosom Res. 2020;138:110232.
87. Gorman G, Toomey E, Flannery C, Redsell S, Hayes C, Huizink A, et al. Fidelity of Interventions to Reduce or Prevent Stress and/or Anxiety from Pregnancy up to Two Years Postpartum: A Systematic Review. Matern Child Health J. 2021;25(2):230-56.
88. Gough N, Brkan L, Subramaniam P, Chiuccariello L, De Petrillo A, Mulsant BH, et al. Feasibility of remotely supervised transcranial direct current stimulation and cognitive remediation: A systematic review. PLoS One. 2020;15(2):e0223029.
89. Gould LF, Dariotis JK, Greenberg MT, Mendelson T. Assessing Fidelity of Implementation (FOI) for School-Based Mindfulness and Yoga Interventions: A Systematic Review. Mindfulness (N Y). 2016;7(1):5-33.
90. Griffin JB, Ridgeway K, Montgomery E, Torjesen K, Clark R, Peterson J, et al. Vaginal ring acceptability and related preferences among women in low- and middle-income countries: A systematic review and narrative synthesis. PLoS One. 2019;14(11):e0224898.
91. Griffiths H. The Acceptability and Feasibility of Using Text Messaging to Support the Delivery of Physical Health Care in those Suffering from a Psychotic Disorder: a Review of the Literature. Psychiatr Q. 2020;91(4):1305-16.
92. Grimshaw SL, Taylor NF, Shields N. The Feasibility of Physical Activity Interventions During the Intense Treatment Phase for Children and Adolescents with Cancer: A Systematic Review. Pediatric blood & cancer. 2016;63(9):1586-93.
93. Gutierrez-Cardona N, Sands P, Roberts G, Lucas JS, Walker W, Salib R, et al. The acceptability and tolerability of nasal douching in children with allergic rhinitis: A systematic review. Int J Pediatr Otorhinolaryngol. 2017;98:126-35.
94. Hadgraft NT, Brakenridge CL, Dunstan DW, Owen N, Healy GN, Lawler SP. Perceptions of the acceptability and feasibility of reducing occupational sitting: review and thematic synthesis. Int J Behav Nutr Phys Act. 2018;15(1):90.
95. Hailemariam M, Bustos T, Montgomery B, Barajas R, Evans LB, Drahota A. Evidence-based intervention sustainability strategies: a systematic review. Implementation science : IS. 2019;14(1):57.
96. Hallam C, Simmonds-Buckley M, Kellett S, Greenhill B, Jones A. The acceptability, effectiveness, and durability of cognitive analytic therapy: Systematic review and meta-analysis. Psychol Psychother. 2021;94 Suppl 1:8-35.
97. Harichund C, Moshabela M. Acceptability of HIV Self-Testing in Sub-Saharan Africa: Scoping Study. AIDS Behav. 2018;22(2):560-8.
98. Hearld LR, Bleser WK, Alexander JA, Wolf LJ. A Systematic Review of the Literature on the Sustainability of Community Health Collaboratives. Med Care Res Rev. 2016;73(2):127-81.
99. Herlitz L, MacIntyre H, Osborn T, Bonell C. The sustainability of public health interventions in schools: a systematic review. Implementation science : IS. 2020;15(1):4.
100. Heynsbergh N, Heckel L, Botti M, Livingston PM. Feasibility, useability and acceptability of technology-based interventions for informal cancer carers: a systematic review. BMC Cancer. 2018;18(1):244.
101. Heywood R, McCarthy AL, Skinner TL. Safety and feasibility of exercise interventions in patients with advanced cancer: a systematic review. Supportive care in cancer : official journal of the Multinational Association of Supportive Care in Cancer. 2017;25(10):3031-50.
102. Hinckley JJ, Douglas NF. Treatment fidelity: its importance and reported frequency in aphasia treatment studies. American journal of speech-language pathology. 2013;22(2):S279-84.
103. Holthe T, Halvorsrud L, Karterud D, Hoel KA, Lund A. Usability and acceptability of technology for community-dwelling older adults with mild cognitive impairment and dementia: a systematic literature review. Clin Interv Aging. 2018;13:863-86.
104. Horrell J, Thompson TP, Taylor AH, Neale J, Husk K, Wanner A, et al. Qualitative systematic review of the acceptability, feasibility, barriers, facilitators and perceived utility of using physical activity in the reduction of and abstinence from alcohol and other drug use. Mental Health and Physical Activity. 2020;19:100355.
105. Hoskins K, Ulrich CM, Shinnick J, Buttenheim AM. Acceptability of financial incentives for health-related behavior change: An updated systematic review. Prev Med. 2019;126:105762.
106. Huang CW, Lee MJ, Wang LJ, Lee PT, Tu YK, Hsu CW, et al. Comparative efficacy and acceptability of treatments for restless legs syndrome in end-stage renal disease: a systematic review and network meta-analysis. Nephrol Dial Transplant. 2020;35(9):1609-18.
107. Ishola F, Cekan J. Evaluating the sustainability of health programmes: A literature review. African Evaluation Journal; Vol 7, No 1 (2019)DO - 104102/aejv7i1369. 2019.
108. Islam MM, Topp L, Day CA, Dawson A, Conigrave KM. The accessibility, acceptability, health impact and cost implications of primary healthcare outlets that target injecting drug users: a narrative synthesis of literature. Int J Drug Policy. 2012;23(2):94-102.
109. Iwelunmor J, Blackstone S, Veira D, Nwaozuru U, Airhihenbuwa C, Munodawafa D, et al. Toward the sustainability of health interventions implemented in sub-Saharan Africa: a systematic review and conceptual framework. Implementation science : IS. 2016;11:43.
110. JaKa MM, Haapala JL, Trapl ES, Kunin-Batson AS, Olson-Bullis BA, Heerman WJ, et al. Reporting of treatment fidelity in behavioural paediatric obesity intervention trials: a systematic review. Obes Rev. 2016;17(12):1287-300.
111. James HM, Papoutsi C, Wherton J, Greenhalgh T, Shaw SE. Spread, Scale-up, and Sustainability of Video Consulting in Health Care: Systematic Review and Synthesis Guided by the NASSS Framework. J Med Internet Res. 2021;23(1):e23775. Med Internet Res. 2021;23(1):e23775.
112. Johnson-Kozlow M, Hovell MF, Rovniak LS, Sirikulvadhana L, Wahlgren DR, Zakarian JM. Fidelity issues in secondhand smoking interventions for children. Nicotine Tob Res. 2008;10(12):1677-90.
113. Jonker LT, Haveman ME, de Bock GH, van Leeuwen BL, Lahr MMH. Feasibility of Perioperative eHealth Interventions for Older Surgical Patients: A Systematic Review. Journal of the American Medical Directors Association. 2020;21(12):1844-51 e2.
114. Kaltenthaler E, Sutcliffe P, Parry G, Beverley C, Rees A, Ferriter M. The acceptability to patients of computerized cognitive behaviour therapy for depression: a systematic review. Psychol Med. 2008;38(11):1521-30.
115. Kayrouz R, Dear BF, Kayrouz B, Karin E, Gandy M, Titov N. Meta-analysis of the efficacy and acceptability of cognitive-behavioural therapy for Arab adult populations experiencing anxiety, depression or post-traumatic stress disorder. Cogn Behav Ther. 2018;47(5):412-30.
116. Keller C, Fleury J, Sidani S, Ainsworth B. Fidelity to Theory in PA Intervention Research. West J Nurs Res. 2009;31(3):289-311.
117. Khanbhai M, Flott K, Darzi A, Mayer E. Evaluating Digital Maturity and Patient Acceptability of Real-Time Patient Experience Feedback Systems: Systematic Review. J Med Internet Res. 2019;21(1):e9076.
118. Kiberu VM, Mars M, Scott RE. Barriers and opportunities to implementation of sustainable e-Health programmes in Uganda: A literature review. Afr J Prim Health Care Fam Med. 2017;9(1):e1-e10.
119. Kininger RL, O'Dell SM, Schultz BK. The Feasibility and Effectiveness of School-Based Modular Therapy: A Systematic Literature Review. School Mental Health. 2018;10(4):339-51.
120. Klausen A, Rohrig R, Lipprandt M. Feasibility of Eyetracking in Critical Care Environments - A Systematic Review. Studies in health technology and informatics. 2016;228:604-8.
121. Kosse NM, Dutmer AL, Dasenbrock L, Bauer JM, Lamoth CJ. Effectiveness and feasibility of early physical rehabilitation programs for geriatric hospitalized patients: a systematic review. BMC geriatrics. 2013;13:107.
122. Krause J, Subklew-Sehume F, Kenyon C, Colebunders R. Acceptability of HIV self-testing: a systematic literature review. BMC Public Health. 2013;13:735.
123. Kriston L, von Wolff A, Westphal A, Holzel LP, Harter M. Efficacy and acceptability of acute treatments for persistent depressive disorder: a network meta-analysis. Depress Anxiety. 2014;31(8):621-30.
124. Lamb R, Miller DE, Lamb R, Akmal T, Hsiao Y-J. Examination of the role of training and fidelity of implementation in the use of assistive communications for children with autism spectrum disorder: a met analysis of the Picture Exchange Communication System. British Journal of Special Education. 2018;45:454-72.
125. Lambert JD, Greaves CJ, Farrand P, Cross R, Haase AM, Taylor AH. Assessment of fidelity in individual level behaviour change interventions promoting physical activity among adults: a systematic review. BMC Public Health. 2017;17(1):765.
126. Lane C, McCrabb S, Nathan N, Naylor PJ, Bauman A, Milat A, et al. How effective are physical activity interventions when they are scaled-up: a systematic review. Int J Behav Nutr Phys Act. 2021;18(1):16.
127. Lauckner C, Whitten P. The State and Sustainability of Telepsychiatry Programs. J Behav Health Serv Res. 2016;43(2):305-18.
128. Learmonth YC, Motl RW. Important considerations for feasibility studies in physical activity research involving persons with multiple sclerosis: a scoping systematic review and case study. Pilot and feasibility studies. 2018;4:1.
129. Lee EKP, Yeung NCY, Xu Z, Zhang D, Yu CP, Wong SYS. Effect and Acceptability of Mindfulness-Based Stress Reduction Program on Patients With Elevated Blood Pressure or Hypertension: A Meta-Analysis of Randomized Controlled Trials. Hypertension. 2020;76(6):1992-2001.
130. Leistikow P, Joseph Cervia M, editors. Acceptability of HIV Testing for Adolescents and Young Adults by Delivery Model: A Systematic Review and Meta-Analysis. Journal of investigative medicine; 2020: 68(4):937-938.
131. Lewis C, Pearce J, Bisson JI. Efficacy, cost-effectiveness and acceptability of self-help interventions for anxiety disorders: systematic review. Br J Psychiatry. 2012;200(1):15-21.
132. Li Y, Coster S, Norman I, Chien WT, Qin J, Ling Tse M, et al. Feasibility, acceptability, and preliminary effectiveness of mindfulness-based interventions for people with recent-onset psychosis: A systematic review. Early Interv Psychiatry. 2021;15(1):3-15.
133. Lieberman-Betz RG. A Systematic Review of Fidelity of Implementation in Parent-Mediated Early Communication Intervention. Topics in Early Childhood Special Education. 2014;35(1):15-27.
134. Liew TM, Lee CS. Reappraising the Efficacy and Acceptability of Multicomponent Interventions for Caregiver Depression in Dementia: The Utility of Network Meta-Analysis. Gerontologist. 2019;59(4):e380-e92.
135. Liptrott S, Bee P, Lovell K. Acceptability of telephone support as perceived by patients with cancer: A systematic review. Eur J Cancer Care (Engl). 2018;27(1).
136. Lovarini M, Clemson L, Dean C. Sustainability of community-based fall prevention programs: a systematic review. J Safety Res. 2013;47:9-17.
137. Lynas J, Hawkins R. Fidelity in school-based child sexual abuse prevention programs: A systematic review. Child Abuse Negl. 2017;72:10-21.
138. MacDonald J, Lorimer K, Knussen C, Flowers P. Interventions to increase condom use among middle-aged and older adults: A systematic review of theoretical bases, behaviour change techniques, modes of delivery and treatment fidelity. J Health Psychol. 2016;21(11):2477-92.
139. Maddocks M, Mockett S, Wilcock A. Is exercise an acceptable and practical therapy for people with or cured of cancer? A systematic review. Cancer Treat Rev. 2009;35(4):383-90.
140. Magalhaes R, Alves J, Thomas RE, Chiaravalloti N, Goncalves OF, Petrosyan A, et al. Are cognitive interventions for multiple sclerosis effective and feasible? Restor Neurol Neurosci. 2014;32(5):623-38.
141. Mahoney C, Veitch L. Interventions for maintaining nasogastric feeding after stroke: An integrative review of effectiveness and acceptability. J Clin Nurs. 2018;27(3-4):e427-e36.
142. Marrazzo JM, Scholes D. Acceptability of urine-based screening for Chlamydia trachomatis in asymptomatic young men: a systematic review. Sex Transm Dis. 2008;35(11 Suppl):S28-33.
143. Marshall S, Vahabi M, Lofters A. Acceptability, Feasibility and Uptake of HPV Self-Sampling Among Immigrant Minority Women: a Focused Literature Review. J Immigr Minor Health. 2019;21(6):1380-93.
144. Mayer KP, Joseph-Isang E, Robinson LE, Parry SM, Morris PE, Neyra JA. Safety and Feasibility of Physical Rehabilitation and Active Mobilization in Patients Requiring Continuous Renal Replacement Therapy: A Systematic Review. Crit Care Med. 2020;48(11):e1112-e20.
145. Maynard BR, Peters KE, Vaughn MG, Sarteschi CM. Fidelity in After-School Program Intervention Research: A Systematic Review. Research on Social Work Practice. 2013;23(6):613-23.
146. McArthur BA, Riosa PB, Preyde M. Treatment fidelity in psychosocial intervention for children and adolescents with comorbid problems. Child Adolesc Ment Health. 2012;17(3):139-45.
147. McCrabb S, Lane C, Hall A, Milat A, Bauman A, Sutherland R, et al. Scaling-up evidence-based obesity interventions: A systematic review assessing intervention adaptations and effectiveness and quantifying the scale-up penalty. Obes Rev. 2019;20(7):964-82.
148. Merz J, Schwarzer G, Gerger H. Comparative Efficacy and Acceptability of Pharmacological, Psychotherapeutic, and Combination Treatments in Adults With Posttraumatic Stress Disorder: A Network Meta-analysis. JAMA Psychiatry. 2019;76(9):904-13.
149. Miller KJ, Adair BS, Pearce AJ, Said CM, Ozanne E, Morris MM. Effectiveness and feasibility of virtual reality and gaming system use at home by older adults for enabling physical activity to improve health-related domains: a systematic review. Age and ageing. 2014;43(2):188-95.
150. Moffa AH, Martin D, Alonzo A, Bennabi D, Blumberger DM, Bensenor IM, et al. Efficacy and acceptability of transcranial direct current stimulation (tDCS) for major depressive disorder: An individual patient data meta-analysis. Prog Neuropsychopharmacol Biol Psychiatry. 2020;99:109836.
151. Mok WKH, R. S, Poh BK, Wee LH, Reilly JJ, Ruzita AT. Sustainability of Childhood Obesity Interventions: A Systematic Review. Pakistan Journal of Nutrition. 2019;18:603-14.
152. Morgan K, Azzani M, Khaing SL, Wong YL, Su TT. Acceptability of Women Self-Sampling versus Clinician-Collected Samples for HPV DNA Testing: A Systematic Review. J Low Genit Tract Dis. 2019;23(3):193-9.
153. Morrell CJ, Sutcliffe P, Booth A, Stevens J, Scope A, Stevenson M, et al. A systematic review, evidence synthesis and meta-analysis of quantitative and qualitative studies evaluating the clinical effectiveness, the cost-effectiveness, safety and acceptability of interventions to prevent postnatal depression. Health Technol Assess. 2016;20(37):1-414.
154. Morris L, Horne M, McEvoy P, Williamson T. Communication training interventions for family and professional carers of people living with dementia: a systematic review of effectiveness, acceptability and conceptual basis. Aging Ment Health. 2018;22(7):863-80.
155. Moug SJ, Bryce A, Mutrie N, Anderson AS. Lifestyle interventions are feasible in patients with colorectal cancer with potential short-term health benefits: a systematic review. Int J Colorectal Dis. 2017;32(6):765-75.
156. Moulton-Perkins A, Moulton D, Cavanagh K, Jozavi A, Strauss C. Systematic review of mindfulness-based cognitive therapy and mindfulness-based stress reduction via group videoconferencing: Feasibility, acceptability, safety, and efficacy [doi:10.1037/int0000216]. US: Educational Publishing Foundation; 2020.
157. Muftin Z, Thompson AR. A systematic review of self-help for disfigurement: effectiveness, usability, and acceptability. Body Image. 2013;10(4):442-50.
158. Murta SG, Sanderson K, Oldenburg B. Process evaluation in occupational stress management programs: a systematic review. Am J Health Promot. 2007;21(4):248-54.
159. Musuuza JS, Barker A, Ngam C, Vellardita L, Safdar N. Assessment of Fidelity in Interventions to Improve Hand Hygiene of Healthcare Workers: A Systematic Review. Infect Control Hosp Epidemiol. 2016;37(5):567-75.
160. Mutz J, Edgcumbe DR, Brunoni AR, Fu CHY. Efficacy and acceptability of non-invasive brain stimulation for the treatment of adult unipolar and bipolar depression: A systematic review and meta-analysis of randomised sham-controlled trials. Neurosci Biobehav Rev. 2018;92:291-303.
161. Mutz J, Vipulananthan V, Carter B, Hurlemann R, Fu CHY, Young AH. Comparative efficacy and acceptability of non-surgical brain stimulation for the acute treatment of major depressive episodes in adults: systematic review and network meta-analysis. BMJ. 2019;364:l1079.
162. Naleppa MJ, Cagle JG. Treatment Fidelity in Social Work Intervention Research: A Review of Published Studies. Research on Social Work Practice. 2010;20(6):674-81.
163. Nawaz A, Skjaeret N, Helbostad JL, Vereijken B, Boulton E, Svanaes D. Usability and acceptability of balance exergames in older adults: A scoping review. Health Informatics J. 2016;22(4):911-31.
164. Neely L, Rispoli M, Gerow S, Hong ER, Hagan-Burke S. Fidelity Outcomes for Autism-Focused Interventionists Coached via Telepractice: a Systematic Literature Review. Journal of Developmental and Physical Disabilities. 2017;29(6):849-74.
165. Nesvag S, McKay JR. Feasibility and Effects of Digital Interventions to Support People in Recovery From Substance Use Disorders: Systematic Review. J Med Internet Res. 2018;20(8):e255.
166. Ngo TD, Park MH, Shakur H, Free C. Comparative effectiveness, safety and acceptability of medical abortion at home and in a clinic: a systematic review. Bull World Health Organ. 2011;89(5):360-70.
167. Njoroge M, Zurovac D, Ogara EA, Chuma J, Kirigia D. Assessing the feasibility of eHealth and mHealth: a systematic review and analysis of initiatives implemented in Kenya. BMC research notes. 2017;10(1):90.
168. O'Connor M, Munnelly A, Whelan R, McHugh L. The Efficacy and Acceptability of Third-Wave Behavioral and Cognitive eHealth Treatments: A Systematic Review and Meta-Analysis of Randomized Controlled Trials. Behav Ther. 2018;49(3):459-75.
169. O'Shea LE, Hawkins JE, Lord J, Schmidt-Hansen M, Hasler E, Cameron S, et al. Access to and sustainability of abortion services: a systematic review and meta-analysis for the National Institute of Health and Care Excellence-new clinical guidelines for England. Hum Reprod Update. 2020;26(6):886-903.
170. Odesanmi TY, Wasti SP, Odesanmi OS, Adegbola O, Oguntuase OO, Mahmood S. Comparative effectiveness and acceptability of home-based and clinic-based sampling methods for sexually transmissible infections screening in females aged 14-50 years: a systematic review and meta-analysis. Sex Health. 2013;10(6):559-69.
171. Okumura Y, Ichikura K. Efficacy and acceptability of group cognitive behavioral therapy for depression: a systematic review and meta-analysis. J Affect Disord. 2014;164:155-64.
172. Padmanathan P, De Silva MJ. The acceptability and feasibility of task-sharing for mental healthcare in low and middle income countries: a systematic review. Soc Sci Med. 2013;97:82-6.
173. Pallas SW, Minhas D, Pérez-Escamilla R, Taylor L, Curry L, Bradley EH. Community health workers in low- and middle-income countries: what do we know about scaling up and sustainability? Am J Public Health. 2013;103(7):e74-82.
174. Parham LD, Cohn ES, Spitzer S, Koomar JA, Miller LJ, Burke JP, et al. Fidelity in sensory integration intervention research. Am J Occup Ther. 2007;61(2):216-27.
175. Patel S, Akhtar A, Malins S, Wright N, Rowley E, Young E, et al. The Acceptability and Usability of Digital Health Interventions for Adults With Depression, Anxiety, and Somatoform Disorders: Qualitative Systematic Review and Meta-Synthesis. J Med Internet Res. 2020;22(7):e16228.
176. Paulauskaite L, Hassiotis A, Ali A. A systematic review of fidelity measurements in complex interventions for people with intellectual disabilities and behaviours that challenge. Advances in Mental Health and Intellectual Disabilities. 2019;13(3/4):158-72.
177. Pellowski J, Mathews C, Kalichman MO, Dewing S, Lurie MN, Kalichman SC. Advancing Partner Notification Through Electronic Communication Technology: A Review of Acceptability and Utilization Research. J Health Commun. 2016;21(6):629-37.
178. Peltea A, Berghea F, Gudu T, Ionescu R. Knee ultrasound from research to real practice: a systematic literature review of adult knee ultrasound assessment feasibility studies. Med Ultrason. 2016;18(4):457-62.
179. Pérez MC, Minoyan N, Ridde V, Sylvestre MP, Johri M. Comparison of registered and published intervention fidelity assessment in cluster randomised trials of public health interventions in low- and middle-income countries: systematic review. Trials. 2018;19(1):410.
180. Peters A, van Driel F, Jansen W. Acceptability of the female condom by sub-Saharan African women: a literature review. Afr J Reprod Health. 2014;18(4):34-44.
181. Pham MD, Agius PA, Romero L, McGlynn P, Anderson D, Crowe SM, et al. Acceptability and feasibility of point-of-care CD4 testing on HIV continuum of care in low and middle income countries: a systematic review. BMC Health Serv Res. 2016;16(a):343.
182. Pierret ACS, Anderson JK, Ford TJ, Burn AM. Review: Education and training interventions, and support tools for school staff to adequately respond to young people who disclose self-harm - a systematic literature review of effectiveness, feasibility and acceptability. Child Adolesc Ment Health. 2020.
183. Preyde M, Burnham PV. Intervention fidelity in psychosocial oncology. J Evid Based Soc Work. 2011;8(4):379-96.
184. Prowse P-T, Nagel T, Meadows G, Enticott J. Treatment Fidelity Over the Last Decade in Psychosocial Clinical Trials Outcome Studies: A Systematic Review. Journal of psychiatry. 2015;18:1-8.
185. Pu J, Zhou X, Liu L, Zhang Y, Yang L, Yuan S, et al. Efficacy and acceptability of interpersonal psychotherapy for depression in adolescents: A meta-analysis of randomized controlled trials. Psychiatry Res. 2017;253:226-32.
186. Qiu D, Hu M, Yu Y, Tang B, Xiao S. Acceptability of psychosocial interventions for dementia caregivers: a systematic review. BMC Psychiatry. 2019;19(1):23.
187. Rehackova L, Arnott B, Araujo-Soares V, Adamson AA, Taylor R, Sniehotta FF. Efficacy and acceptability of very low energy diets in overweight and obese people with Type 2 diabetes mellitus: a systematic review with meta-analyses. Diabet Med. 2016;33(5):580-91.
188. Reilly R, Evans K, Gomersall J, Gorham G, Peters MD, Warren S, et al. Effectiveness, cost effectiveness, acceptability and implementation barriers/enablers of chronic kidney disease management programs for Indigenous people in Australia, New Zealand and Canada: a systematic review of mixed evidence. BMC Health Serv Res. 2016;16:119.
189. Ridgers ND, McNarry MA, Mackintosh KA. Feasibility and Effectiveness of Using Wearable Activity Trackers in Youth: A Systematic Review. JMIR Mhealth Uhealth. 2016;4(4):e129.
190. Rismawan W, Marchira CR, Rahmat I. Usability, Acceptability, and Adherence Rates of Mobile Application Interventions for Prevention or Treatment of Depression: A Systematic Review. J Psychosoc Nurs Ment Health Serv. 2021;59(2):41-7.
191. Robinson L, Hutchings D, Corner L, Beyer F, Dickinson H, Vanoli A, et al. A systematic literature review of the effectiveness of non-pharmacological interventions to prevent wandering in dementia and evaluation of the ethical implications and acceptability of their use. Health Technol Assess. 2006;10(26):iii, ix-108.
192. Robinson L, Hutchings D, Dickinson HO, Corner L, Beyer F, Finch T, et al. Effectiveness and acceptability of non-pharmacological interventions to reduce wandering in dementia: a systematic review. Int J Geriatr Psychiatry. 2007;22(1):9-22.
193. Rojas-Andrade R, Bahamondes LL. Is Implementation Fidelity Important? A Systematic Review on School-Based Mental Health Programs. Contemporary School Psychology. 2019;23(4):339-50.
194. Rus-Calafell M, Garety P, Sason E, Craig TJK, Valmaggia LR. Virtual reality in the assessment and treatment of psychosis: a systematic review of its utility, acceptability and effectiveness. Psychol Med. 2018;48(3):362-91.
195. Ruzicka M, Hiremath S, Steiner S, Helis E, Szczotka A, Baker P, et al. What is the feasibility of implementing effective sodium reduction strategies to treat hypertension in primary care settings? A systematic review. Journal of hypertension. 2014;32(7):1388-94; discussion 94.
196. Salamh P, Cook C, Reiman MP, Sheets C. Treatment effectiveness and fidelity of manual therapy to the knee: A systematic review and meta-analysis. Musculoskeletal Care. 2017;15(3):238-48.
197. Salamh PA, Liu X, Hanney WJ, Sprague PA, Kolber MJ. The efficacy and fidelity of clinical interventions used to reduce posterior shoulder tightness: a systematic review with meta-analysis. J Shoulder Elbow Surg. 2019;28(6):1204-13.
198. Samara MT, Huhn M, Chiocchia V, Schneider-Thoma J, Wiegand M, Salanti G, et al. Efficacy, acceptability, and tolerability of all available treatments for insomnia in the elderly: a systematic review and network meta-analysis. Acta Psychiatr Scand. 2020;142(1):6-17.
199. Schaap R, Bessems K, Otten R, Kremers S, van Nassau F. Measuring implementation fidelity of school-based obesity prevention programmes: a systematic review. Int J Behav Nutr Phys Act. 2018;15(1):75.
200. Schinckus L, Van den Broucke S, Housiaux M. Assessment of implementation fidelity in diabetes self-management education programs: a systematic review. Patient Educ Couns. 2014;96(1):13-21.
201. Schober I, Sharpe H, Schmidt U. The reporting of fidelity measures in primary prevention programmes for eating disorders in schools. Eur Eat Disord Rev. 2013;21(5):374-81.
202. Scholz L, Werle J, Philipsen A, Schulze M, Collonges J, Gensichen J. Effects and feasibility of psychological interventions to reduce inattention symptoms in adults with ADHD: a systematic review. Journal of mental health (Abingdon, England). 2020:1-14.
203. Schroder J, Truijen S, Van Criekinge T, Saeys W. Feasibility and effectiveness of repetitive gait training early after stroke: A systematic review and meta-analysis. Journal of rehabilitation medicine. 2019;51(2):78-88.
204. Schroder J, van Criekinge T, Embrechts E, Celis X, Van Schuppen J, Truijen S, et al. Combining the benefits of tele-rehabilitation and virtual reality-based balance training: a systematic review on feasibility and effectiveness. Disability and rehabilitation Assistive technology. 2019;14(1):2-11.
205. Scope A, Uttley L, Sutton A. A qualitative systematic review of service user and service provider perspectives on the acceptability, relative benefits, and potential harms of art therapy for people with non-psychotic mental health disorders. Psychol Psychother. 2017;90(1):25-43.
206. Scott RL, Cummings GE, Newburn-Cook C. The feasibility and effectiveness of emergency department based hypertension screening: a systematic review. Journal of the American Academy of Nurse Practitioners. 2011;23(9):493-500.
207. Seitz DP, Brisbin S, Herrmann N, Rapoport MJ, Wilson K, Gill SS, et al. Efficacy and feasibility of nonpharmacological interventions for neuropsychiatric symptoms of dementia in long term care: a systematic review. Journal of the American Medical Directors Association. 2012;13(6):503-6 e2.
208. Sheffield KM, Woods-Giscombe CL. Efficacy, Feasibility, and Acceptability of Perinatal Yoga on Women's Mental Health and Well-Being: A Systematic Literature Review. J Holist Nurs. 2016;34(1):64-79.
209. Shek AC, Biondi A, Ballard D, Wykes T, Simblett SK. Technology-based interventions for mental health support after stroke: A systematic review of their acceptability and feasibility. Neuropsychol Rehabil. 2021;31(3):432-52.
210. Shrime MG, Sleemi A, Ravilla TD. Charitable platforms in global surgery: a systematic review of their effectiveness, cost-effectiveness, sustainability, and role training. World J Surg. 2015;39(1):10-20.
211. Simmonds-Buckley M, Bennion MR, Kellett S, Millings A, Hardy GE, Moore RK. Acceptability and Effectiveness of NHS-Recommended e-Therapies for Depression, Anxiety, and Stress: Meta-Analysis. J Med Internet Res. 2020;22(10):e17049.
212. Simmonds-Buckley M, Kellett S, Waller G. Acceptability and Efficacy of Group Behavioral Activation for Depression Among Adults: A Meta-Analysis. Behav Ther. 2019;50(5):864-85.
213. Simon N, McGillivray L, Roberts NP, Barawi K, Lewis CE, Bisson JI. Acceptability of internet-based cognitive behavioural therapy (i-CBT) for post-traumatic stress disorder (PTSD): a systematic review. Eur J Psychotraumatol. 2019;10(1):1646092.
214. Singh B, Hayes SC, Spence RR, Steele ML, Millet GY, Gergele L. Exercise and colorectal cancer: a systematic review and meta-analysis of exercise safety, feasibility and effectiveness. Int J Behav Nutr Phys Act. 2020;17(1):122.
215. Singh B, Spence R, Steele ML, Hayes S, Toohey K. Exercise for Individuals With Lung Cancer: A Systematic Review and Meta-Analysis of Adverse Events, Feasibility, and Effectiveness. Seminars in oncology nursing. 2020;36(5):151076.
216. Singh B, Spence RR, Steele ML, Sandler CX, Peake JM, Hayes SC. A Systematic Review and Meta-Analysis of the Safety, Feasibility, and Effect of Exercise in Women With Stage II+ Breast Cancer. Archives of physical medicine and rehabilitation. 2018;99(12):2621-36.
217. Sjostrom S, Dragoman M, Fonhus MS, Ganatra B, Gemzell-Danielsson K. Effectiveness, safety, and acceptability of first-trimester medical termination of pregnancy performed by non-doctor providers: a systematic review. BJOG. 2017;124(13):1928-40.
218. Skea ZC, Aceves-Martins M, Robertson C, De Bruin M, Avenell A, team R. Acceptability and feasibility of weight management programmes for adults with severe obesity: a qualitative systematic review. BMJ open. 2019;9(9):e029473.
219. Solmi M, Wade TD, Byrne S, Del Giovane C, Fairburn CG, Ostinelli EG, et al. Comparative efficacy and acceptability of psychological interventions for the treatment of adult outpatients with anorexia nervosa: a systematic review and network meta-analysis. Lancet Psychiatry. 2021;8(3):215-24.
220. Soneson E, Howarth E, Ford T, Humphrey A, Jones PB, Thompson Coon J, et al. Feasibility of School-Based Identification of Children and Adolescents Experiencing, or At-risk of Developing, Mental Health Difficulties: a Systematic Review. Prevention science : the official journal of the Society for Prevention Research. 2020;21(5):581-603.
221. Sotirova MB, McCaughan EM, Ramsey L, Flannagan C, Kerr DP, O'Connor SR, et al. Acceptability of online exercise-based interventions after breast cancer surgery: systematic review and narrative synthesis. J Cancer Surviv. 2021;15(2):281-310.
222. Sprogis SK, Currey J, Considine J. Patient acceptability of wearable vital sign monitoring technologies in the acute care setting: A systematic review. J Clin Nurs. 2019;28(15-16):2732-44.
223. Stephen C, McInnes S, Halcomb E. The feasibility and acceptability of nurse-led chronic disease management interventions in primary care: An integrative review. J Adv Nurs. 2018;74(2):279-88.
224. Strauss C, Thomas N, Hayward M. Can we respond mindfully to distressing voices? A systematic review of evidence for engagement, acceptability, effectiveness and mechanisms of change for mindfulness-based interventions for people distressed by hearing voices. Front Psychol. 2015;6:1154.
225. Taleghani S, Joseph-Davey D, West SB, Klausner HJ, Wynn A, Klausner JD. Acceptability and efficacy of partner notification for curable sexually transmitted infections in sub-Saharan Africa: A systematic review. Int J STD AIDS. 2019;30(3):292-303.
226. Tarabay R, El Rassi R, Dakik A, Harb A, Ballout RA, Diab B, et al. Knowledge, attitudes, beliefs, values, preferences, and feasibility in relation to the use of injection safety devices in healthcare settings: a systematic review. Health Qual Life Outcomes. 2016;14:102.
227. Taylor PJ, Thompson CH, Brinkworth GD. Effectiveness and acceptability of continuous glucose monitoring for type 2 diabetes management: A narrative review. J Diabetes Investig. 2018;9(4):713-25.
228. Terrens AF, Soh SE, Morgan PE. The efficacy and feasibility of aquatic physiotherapy for people with Parkinson's disease: a systematic review. Disability and rehabilitation. 2018;40(24):2847-56.
229. Toh HM, Ghazali SE, Subramaniam P. The Acceptability and Usefulness of Cognitive Stimulation Therapy for Older Adults with Dementia: A Narrative Review. Int J Alzheimers Dis. 2016;2016:5131570.
230. Toomey E, Currie-Murphy L, Matthews J, Hurley DA. Implementation fidelity of physiotherapist-delivered group education and exercise interventions to promote self-management in people with osteoarthritis and chronic low back pain: a rapid review part II. Man Ther. 2015;20(2):287-94.
231. Toomey E, Matvienko-Sikar K, Heary C, Delaney L, Queally M, Hayes CB, et al. Intervention Fidelity Within Trials of Infant Feeding Behavioral Interventions to Prevent Childhood Obesity: A Systematic Review. Ann Behav Med. 2019;53(1):75-97.
232. Tough D, Robinson J, Gowling S, Raby P, Dixon J, Harrison SL. The feasibility, acceptability and outcomes of exergaming among individuals with cancer: a systematic review. BMC Cancer. 2018;18(1):1151.
233. Troup J, Fuhr DC, Woodward A, Sondorp E, Roberts B. Barriers and facilitators for scaling up mental health and psychosocial support interventions in low- and middle-income countries for populations affected by humanitarian crises: a systematic review. Int J Ment Health Syst. 2021;15(1):5.
234. Troy V, McPherson KE, Emslie C, Gilchrist E. The Feasibility, Appropriateness, Meaningfulness, and Effectiveness of Parenting and Family Support Programs Delivered in the Criminal Justice System: A Systematic Review. Journal of child and family studies. 2018;27(6):1732-47.
235. Turrini G, Purgato M, Acarturk C, Anttila M, Au T, Ballette F, et al. Efficacy and acceptability of psychosocial interventions in asylum seekers and refugees: systematic review and meta-analysis. Epidemiol Psychiatr Sci. 2019;28(4):376-88.
236. Vassall A, Compernolle P. Estimating the resource needs of scaling-up HIV/AIDS and tuberculosis interventions in sub-Saharan Africa: a systematic review for national policy makers and planners. Health Policy. 2006;79(1):1-15.
237. Wang Z, Zhang Q, Huang H, Liu Z. The efficacy and acceptability of curcumin for the treatment of depression or depressive symptoms: A systematic review and meta-analysis. J Affect Disord. 2021;282:242-51.
238. Weber M, Belala N, Clemson L, Boulton E, Hawley-Hague H, Becker C, et al. Feasibility and Effectiveness of Intervention Programmes Integrating Functional Exercise into Daily Life of Older Adults: A Systematic Review. Gerontology. 2018;64(2):172-87.
239. Westercamp N, Bailey RC. Acceptability of male circumcision for prevention of HIV/AIDS in sub-Saharan Africa: a review. AIDS Behav. 2007;11(3):341-55.
240. Wiessing L, Ferri M, Grady B, Kantzanou M, Sperle I, Cullen KJ, et al. Hepatitis C virus infection epidemiology among people who inject drugs in Europe: a systematic review of data for scaling up treatment and prevention. PLoS One. 2014;9(7):e103345.
241. Woodnutt S. Is Lean sustainable in today's NHS hospitals? A systematic literature review using the meta-narrative and integrative methods. Int J Qual Health Care. 2018;30(8):578-86.
242. Wright C, Catty J, Watt H, Burns T. A systematic review of home treatment services--classification and sustainability. Soc Psychiatry Psychiatr Epidemiol. 2004;39(10):789-96.
243. Yang L, Zhou X, Pu J, Liu L, Cuijpers P, Zhang Y, et al. Efficacy and acceptability of psychological interventions for social anxiety disorder in children and adolescents: a meta-analysis of randomized controlled trials. Eur Child Adolesc Psychiatry. 2019;28(1):79-89.
244. Yang L, Zhou X, Zhou C, Zhang Y, Pu J, Liu L, et al. Efficacy and Acceptability of Cognitive Behavioral Therapy for Depression in Children: A Systematic Review and Meta-analysis. Acad Pediatr. 2017;17(1):9-16.
245. Yeary KH, Klos LA, Linnan L. The examination of process evaluation use in church-based health interventions: a systematic review. Health Promot Pract. 2012;13(4):524-34.
246. Yin B, Teng T, Tong L, Li X, Fan L, Zhou X, et al. Efficacy and acceptability of parent-only group cognitive behavioral intervention for treatment of anxiety disorder in children and adolescents: a meta-analysis of randomized controlled trials. BMC Psychiatry. 2021;21(1):29.
247. Yuan S, Zhou X, Zhang Y, Zhang H, Pu J, Yang L, et al. Comparative efficacy and acceptability of bibliotherapy for depression and anxiety disorders in children and adolescents: a meta-analysis of randomized clinical trials. Neuropsychiatr Dis Treat. 2018;14:353-65.
248. Zaga CJ, Berney S, Vogel AP. The Feasibility, Utility, and Safety of Communication Interventions With Mechanically Ventilated Intensive Care Unit Patients: A Systematic Review. American journal of speech-language pathology. 2019;28(3):1335-55.
249. Zhang H, Zhang Y, Yang L, Yuan S, Zhou X, Pu J, et al. Efficacy and Acceptability of Psychotherapy for Anxious Young Children: A Meta-analysis of Randomized Controlled Trials. J Nerv Ment Dis. 2017;205(12):931-41.
250. Zhou X, Hetrick SE, Cuijpers P, Qin B, Barth J, Whittington CJ, et al. Comparative efficacy and acceptability of psychotherapies for depression in children and adolescents: A systematic review and network meta-analysis. World Psychiatry. 2015;14(2):207-22.
251. Zhou X, Teng T, Zhang Y, Del Giovane C, Furukawa TA, Weisz JR, et al. Comparative efficacy and acceptability of antidepressants, psychotherapies, and their combination for acute treatment of children and adolescents with depressive disorder: a systematic review and network meta-analysis. Lancet Psychiatry. 2020;7(7):581-601.
252. Zimmermann FF, Burrell B, Jordan J. The acceptability and potential benefits of mindfulness-based interventions in improving psychological well-being for adults with advanced cancer: A systematic review. Complement Ther Clin Pract. 2018;30:68-78.
